# Liposomal Permeation Assay for Droplet-Scale Pharmacokinetic Screening

**DOI:** 10.1021/acs.jmedchem.3c00138

**Published:** 2023-04-19

**Authors:** Juan Hu, Alix I Chan, Emel Adaligil, Ivy Kekessie, Mifune Takahashi, Aimin Song, Christian N. Cunningham, Brian M. Paegel

**Affiliations:** † Department of Pharmaceutical Sciences, University of California, Irvine, California 92617, United States; ‡ Department of Peptide Therapeutics, Genentech Inc., South San Francisco, California 94080, United States; § Department of Drug Metabolism and Pharmacokinetics, Genentech Inc., South San Francisco 94080, United States; ∥ Departments of Chemistry and Biomedical Engineering, University of California, Irvine, California 92617, United States

## Abstract

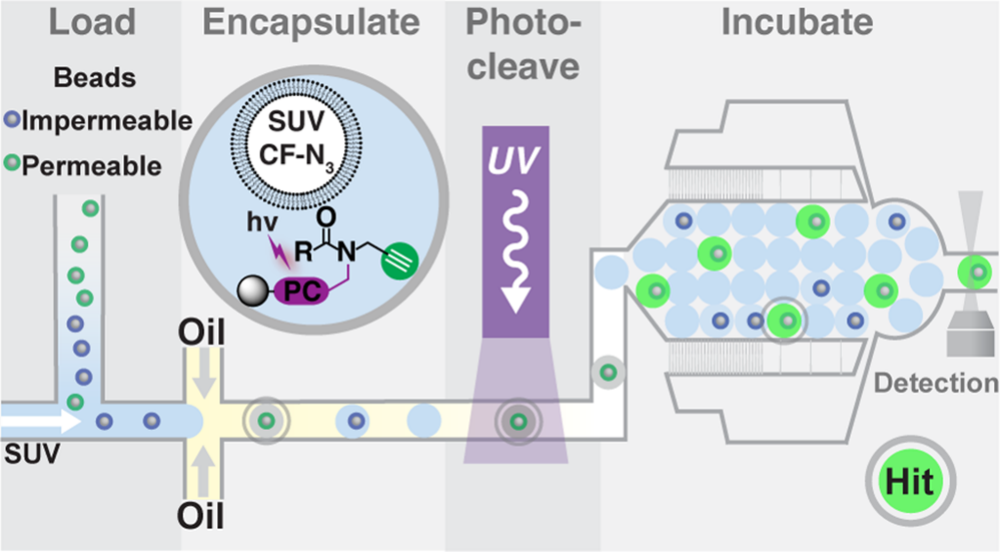

Combinatorial library screening increasingly explores chemical space beyond the Ro5 (bRo5), which is useful for investigating ”undruggable” targets but suffers compromised cellular permeability and therefore bioavailability. Moreover, structure–permeation relationships for bRo5 molecules are unclear partially because high-throughput permeation measurement technology for encoded combinatorial libraries is still nascent. Here, we present a permeation assay that is scalable to combinatorial library screening. A liposomal fluorogenic azide probe transduces permeation of alkyne-labeled molecules into small unilamellar vesicles via copper-catalyzed azide–alkyne cycloaddition. Control alkynes (e.g., propargylamine, various alkyne-labeled PEGs) benchmarked the assay. Cell-permeable macrocyclic peptides, exemplary bRo5 molecules, were alkyne labeled and shown to retain permeability. The assay was miniaturized to microfluidic droplets with high assay quality (*Z*′ ≥ 0.5), demonstrating excellent discrimination of photocleaved known membrane-permeable and -impermeable model library beads. Droplet-scale permeation screening will enable pharmacokinetic mapping of bRo5 libraries to build predictive models.

## Introduction

Lipinski’s Rule of 5 (Ro5) describes a set of physicochemical properties that collectively correlate small molecule structure with oral bioavailability and has strongly directed drug discovery and development over the past decades. The Ro5^1^ properties comprise molecular weight (MW < 500 Da), hydrogen bond donor/acceptor count (HBD/HBA <5/<10), and lipophilicity (cLogP < 5); later studies further implicated molecular permeation (*P*_e_ > 10^–6^ cm/s).^2^ These parameters precipitated the classification of protein targets as “undruggable” if an Ro5-compliant molecule would not be predicted to bind and modulate the target’s activity. These difficult targets, such as protein–protein interfaces^3^ and intrinsically disordered proteins,^4^ account for the vast majority of the human proteome and therefore interest in investigating them is accelerating.^5,6^ Exploring molecules with higher MW (500–1500 Da) and HBD/HBA (>5/>10) can enable chemical probe discovery,^7,8^ however, chemical matter that is beyond the Rule of 5 (bRo5) criteria usually exhibits compromised cell permeation and therefore bioavailability.^7,9,10^ In this bRo5 chemical space, a central remaining correlate with bioavailability short of animal studies is membrane permeability.

Membrane permeability is typically determined using two different transwell assays. The parallel artificial membrane permeability assay (PAMPA) measures the membrane permeation property of molecules via passive diffusion through a synthetic lipid membrane,^11^ and the cellular monolayer absorption assay measures permeation through a confluent layer of either MDCK (Madin-Darby canine kidney) or Caco-2 (human colon carcinoma) cells.^12,13^ In both assays, the permeating molecule is detected via LC-MS or UV absorption. Neither transwell assay is particularly convenient for ultrahigh-throughput screening, but they have been deployed on the scale of conventional compound collections because knowledge of membrane permeability is critical for further development.^2^ Recent innovative permeation measurement technology development has included fluorogenic copper-catalyzed azide alkyne cycloaddition (CuAAC),^14^ liposomal dye displacement or LC-MS liposome pulldown,^15−20^ droplet-interface bilayer (DIB) permeation assay,^21,22^ black lipid membrane (BLM) label-free penetration assay,^23^ or cellular chloroalkane penetration assay (CAPA).^24,25^ Although all of these approaches can measure the permeation of bRo5 molecules individually, none are amenable to screening large split-and-pool combinatorial libraries (>10^4^ members), which are the source of most bRo5 chemical matter.

Combinatorial library synthesis affords the opportunity to explore novel bRo5 chemical space cheaply and expansively. In particular, encoded library modalities, such as DNA-encoded library (DEL) technology and the mRNA display-based random nonstandard peptides integrated discovery (RaPID) platform, enable synthesis and affinity selection of billion-member compound collections.^26−30^ Unlike conventional compound collections, which can be analyzed for bioavailability by proxy using assays of membrane permeation, the inherent nature of encoded libraries as complex mixtures renders them incompatible. As a result, almost nothing is known about the pharmacokinetic (PK) properties of these libraries, and very recent disclosures have detailed how combinatorial split-and-pool libraries can be analyzed in pools via permeation assays to identify structure–permeability trends, highlighting the growing interest and need for technology development in this area.^31,32^ Solid-phase one-bead-one-compound (OBOC) DEL synthesis protocols^33−35^ and accompanying microfluidic activity-based screening instrumentation^36,37^ have expanded library analysis beyond binding. We desired to integrate known droplet-scale permeation measurement approaches^38,39^ with innovations in homogeneous fluorescence-based PAMPA to enable combinatorial library-scale analysis and thereby uncover the molecular determinants of permeability in bRo5 chemical space.

Here, we disclose a liposomal permeation assay that is scalable to and compatible with high-throughput combinatorial chemical library functional screening. We posited that small unilamellar vesicle (SUV, ∼0.1 μm diameter) liposomes would provide model bilayer membranes for assaying permeation and that SUVs would be advantageous for droplet-based screening.^16,39^ A fluorogenic CuAAC probe is encapsulated in the SUVs, which transduce the permeation of an alkyne-labeled molecule of interest into a gain of fluorescence signal. We benchmark the assay in microtiter plate format using alkynes with known permeation (propargylamine, PEG) and known cell-permeable alkyne-tagged macrocyclic peptides as model bRo5 combinatorial library members. The validated assay was further miniaturized to microfluidic droplets to demonstrate compatibility with high-throughput OBOC library screening.

## Results and Discussion

### Development of a Fluorogenic Liposomal Permeation Assay

**Figure 1:**
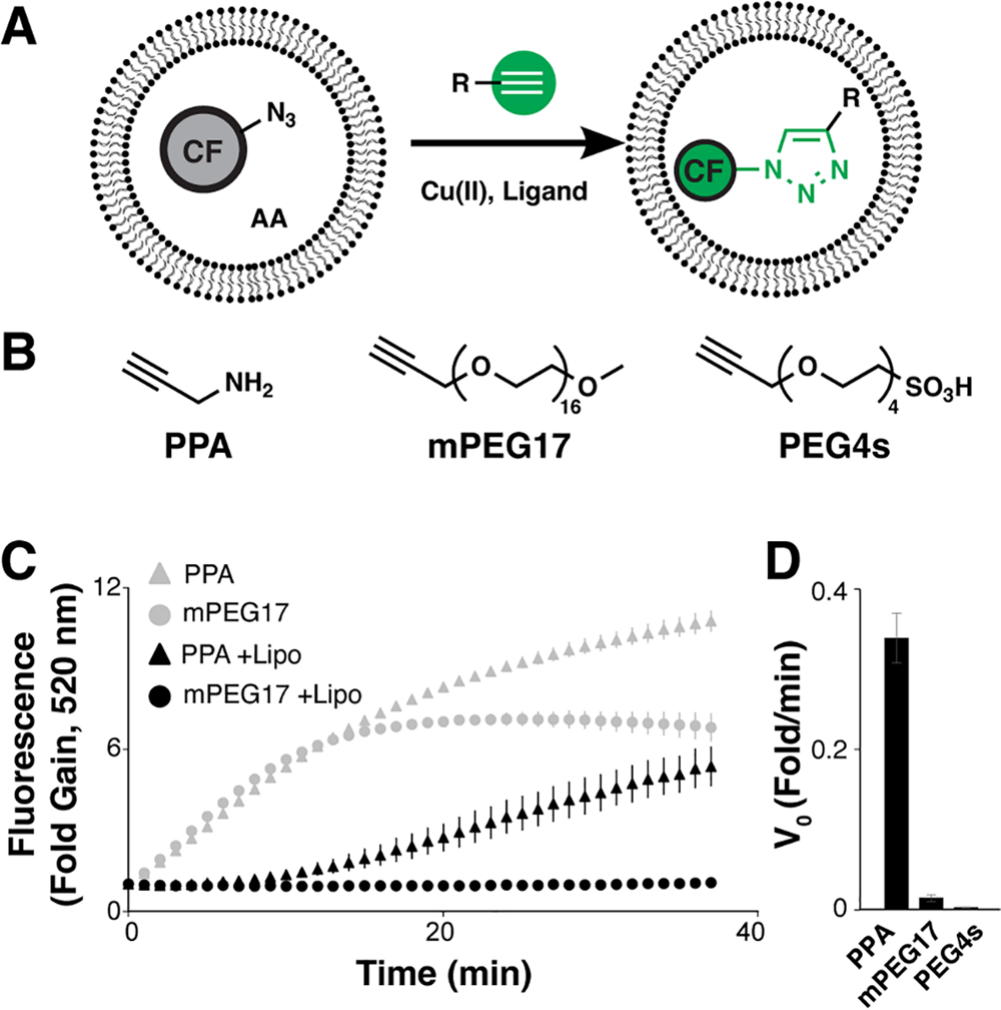
Liposomal permeation assay. (A) Membrane-impermeable fluorogenic azide probe (CF-N_3_) is encapsulated with ascorbic acid (AA) in small unilamellar vesicles. Probe fluorescence increases only upon reaction with membrane-permeable alkyne (green) via CuAAC in the presence of Cu(I) and TBTA ligand. (B) Control alkynes for assay development include: propargylamine (PPA), propargyl m-PEG17 (mPEG17), and propargyl-PEG4-sulfonic acid (PEG4s). (C) Fluorogenic CuAAC reaction progress with CF-N_3_ and liposomal CF-N_3_ (+Lipo) using PPA and PEG17 was measured as the fold gain in fluorescence over the initial value. (D) Initial CuAAC reaction velocity (fold gain/min) was calculated to quantitate permeation rate. Error bars indicate the standard error of the mean (*N* = 3).

Liposomes were explored as a model bilayer membrane for measuring permeation. SUVs (∼100 nm diameter) were loaded with membrane-impermeable fluorogenic azide probe (CalFluor 488, CF-N_3_)^40^ and ascorbic acid (AA) during lipid film hydration. Unencapsulated probe was removed by dialysis after extrusion. SUV formation and uniformity were confirmed via dynamic light scattering (DLS, Supporting Information (SI), Figure S1). Only membrane-permeable alkynes penetrate the SUV bilayer, reacting with the CF-N_3_ probe in a CuAAC reaction, resulting in a fluorescent triazole product (Figure 1A). Alkyne-tagged permeation controls (Figure 1B) included propargylamine (PPA, permeable), propargyl-PEG17-methane (mPEG17, impermeable), and propargyl-PEG4-sulfonic acid (PEG4s, impermeable). The control alkynes’ permeabilities were measured previously^38^ or via PAMPA (SI, Table S1). Controls were analyzed in real-time fluorogenic CuAAC reactions in the presence of CF-N_3_-loaded DOPC-POPC SUVs or with free CF-N_3_ (Figure 1C) and the initial CuAAC reaction velocity (*V*_0_, fold/min) was extracted to quantitate permeation (Figure 1D) and, ultimately, statistical assay performance, *Z*′.^41^ The *Z*′ for PPA/mPEG17 and PPA/PEG4s was 0.66 and 0.71, respectively.

Control alkyne permeation in the liposomal assay tracked with gold-standard PAMPA permeation measurements. All alkynes reacted readily with free CF-N_3_, but only membrane-permeable PPA reacted with the liposomal CF-N_3_. The *V*_0_ values for reaction of PPA and mPEG17 with free CF-N_3_ were statistically indistinguishable. Reaction of PPA with liposomal CF-N_3_ resulted in a ∼10 min lag before reaching steady *V*_0_. Reaction of mPEG17 with liposomal CF-N_3_ was undetectable compared to PPA reaction with liposomal CF-N_3_ and to mPEG17 reaction with free CF-N_3_. We attribute this behavior to the impermeability of mPEG17 (PAMPA *P*_e_ > 4 × 10^–9^cm/s). High *Z*′ values, reflecting excellent assay quality, substantiated the assay’s suitability for implementation in microplate- and microfluidic droplet-scale HTS.

**Figure 2:**
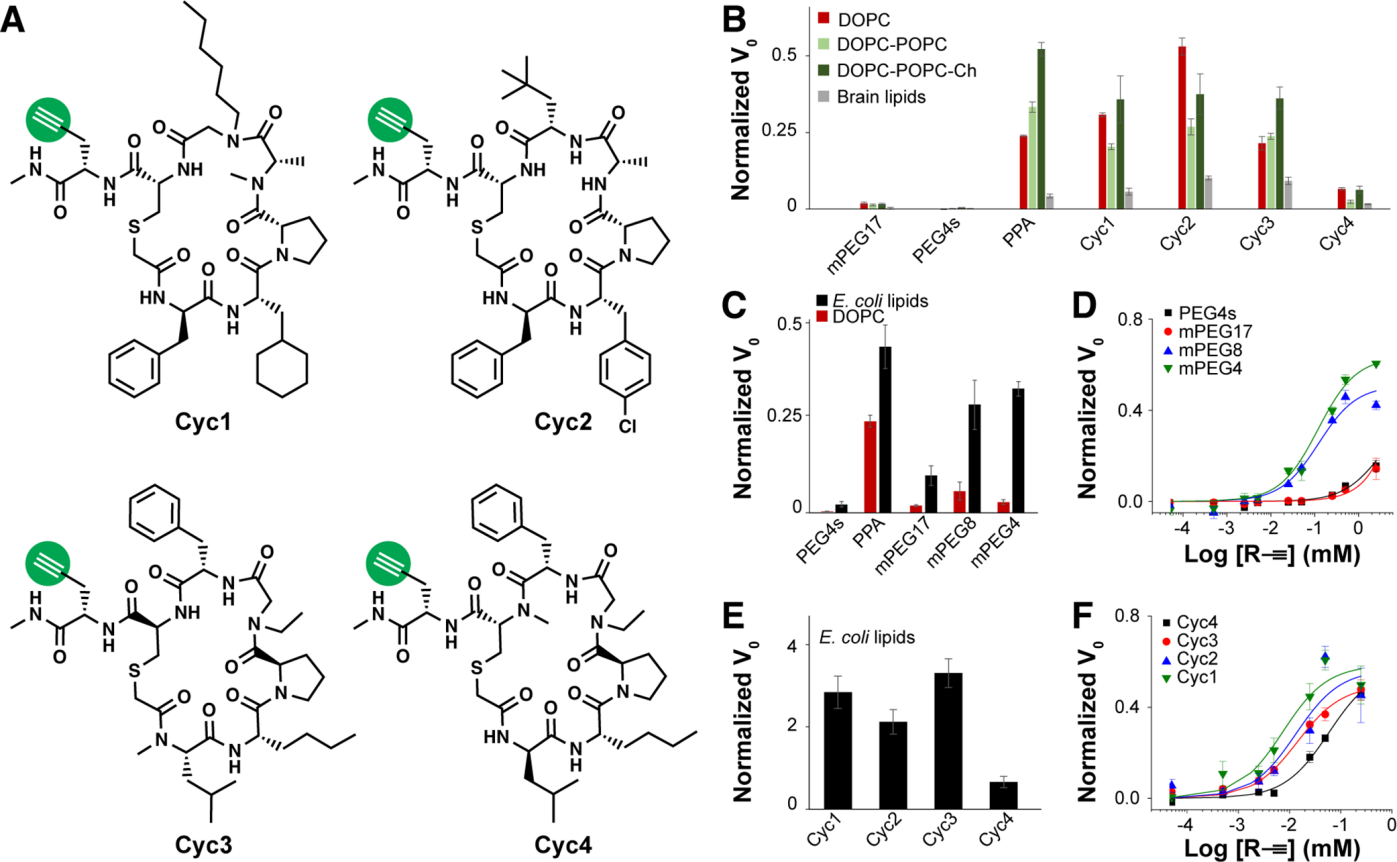
Liposomal permeation assay using different lipid mixtures. (A) Various cell-permeable macrocycles were alkyne-labeled (Cyc1–4). (B) Permeation rates of mPEG17, PEG4s, PPA, and Cyc1–4 were measured using liposomes composed of DOPC, DOPC/POPC, DOPC/POPC/Ch (20 mol % Ch), and brain polar lipids. Initial CuAAC reaction velocity (*V*_0_, fold/min) in the liposomal assay was normalized to the corresponding reaction velocity without liposomes. (C) mPEG17, PEG4s, PPA, mPEG8, and mPEG4 were tested in liposomal permeation assays of *E. coli* extract polar lipids and DOPC, and (D) concentration-dependent permeation was measured. (E) Cyc1–4 were tested in liposomal permeation assays of *E. coli* extract polar lipids, and further measured at varied concentrations in the assay (F). Error bars indicate the standard error of the mean (*N* = 3).

PAMPA is compatible with different lipid bilayer compositions and permeating molecules, thus we sought to determine whether the liposomal CuAAC permeation assay exhibited similar versatility. A series of membrane-permeable bRo5 macrocyclic peptides was derivatized by addition of a C-terminal propargylglycine residue to introduce the requisite alkyne label (Cyc1–4, Figure 2A; SI, Figure S2). The four macrocycles’ permeation coefficients as determined via PAMPA were largely independent of alkyne labeling (*P*_e_; SI, Table S1). Alkyne-labeled macrocycle permeation was measured in the liposomal permeation assay using DOPC, DOPC-POPC, DOPC-POPC-cholesterol (DOPC-POPC-Ch), and brain polar lipids and compared with permeation positive and negative controls, PPA and PEG4s, respectively (Figure 2B; SI, Figure S3). Molecular weight correlation with permeability was explored next using a model series of alkyne-labeled PEG (mPEG17, mPEG8, mPEG4) in either single-concentration (Figure 2C) or concentration-dependent permeation assays (Figure 2D) together with a bRo5 macrocycle panel (Figure 2E,F). Concentration-dependent permeation measurement inflection points, termed “PC50,” were tabulated for all control and peptide macrocycles (SI, Table S1). As an example of the assay’s ability to detect permeation of an alternative alkyne-labeled scaffold, we synthesized and assayed the cell-penetrating peptide ”DD5o”^24^ and two of its diastereomers. DD5o was also observed to be permeable in the liposomal permeation assay, while stereochemical inversions altered permeation to varying degrees (SI, Figure S5).

The liposomal permeation assay accommodated structurally divergent alkyne-tagged molecules and expected permeation trends were preserved using compositionally diverse SUVs. Several macrocyclic peptides that model those typically found in display-type encoded libraries were synthesized and evaluated in both PAMPA and liposomal permeation assays. The macrocycles contain a thioether linkage, a variety of natural and non-natural amino acids, points of stereochemical inversion, and diverse *N*-methylations. These features are variously known to influence compound stability and permeability, and these four compounds were selected from a larger library of macrocycles based on their observed and enhanced permeability.^42^ Lipid formulation was generally important across all alkynes: addition of cholesterol to DOPC-POPC bilayers increased permeation of all species, in agreement with one known behavior of cholesterol in bilayers^43^ and brain lipid SUVs were less permeable for all species studied. Permeation positive and negative controls (PPA and mPEG17/PEG4s, respectively) were consistent across the different lipid formulations and Cyc1–3 all exhibited robust permeation, in agreement with their high PAMPA *P*_e_ compared to the more modest *P*_e_ of Cyc4. Membrane-permeable species were readily detected regardless of lipid formulation, although some membrane formulations were more permeable than others. Permeation also trended with expectations based on the known inverse correlation between molecular weight and permeability: the more massive mPEG17 was largely impermeable, while the Ro5-compliant mPEG4 and mPEG8 readily permeated through multiple SUV formulations. The liposomal permeation measurement of the DD5o cell-penetrating peptide agrees with Kritzer’s observations using CAPA.^24^ The unusual dependence of permeation on stereochemical configuration agrees with ours and others’ findings; further synthesis and assay data are needed to understand these structure-permeation trends.^32,38,44^

To quantify permeation analogous to the permeation coefficient, *P*_e_, we devised the PC50 measurement. The *P*_e_ is formally an apparent diffusion coefficient, where passive diffusion through the membrane is assumed to be limiting for permeation. The PC50 measurement detects whether saturating permeation occurred. At this concentration, the fluorogenic CuAAC reaction progress is no longer limited by permeation through the membrane. PC50 values, determined for *E. coli* extract SUVs, correlated with PAMPA *P*_e_ values. Thus, PC50 may be a reasonable proxy for the more standard measurement. End point assays are nonetheless more practical for medium- and high-throughput screening in microplates without the need for transwell assemblies, LC-MS sample detection, or engineered cell lines, and the liposomal assay’s performance is sufficient for these purposes.

### Microfluidic Droplet-Scale Liposomal Permeation Assay

**Figure 3:**
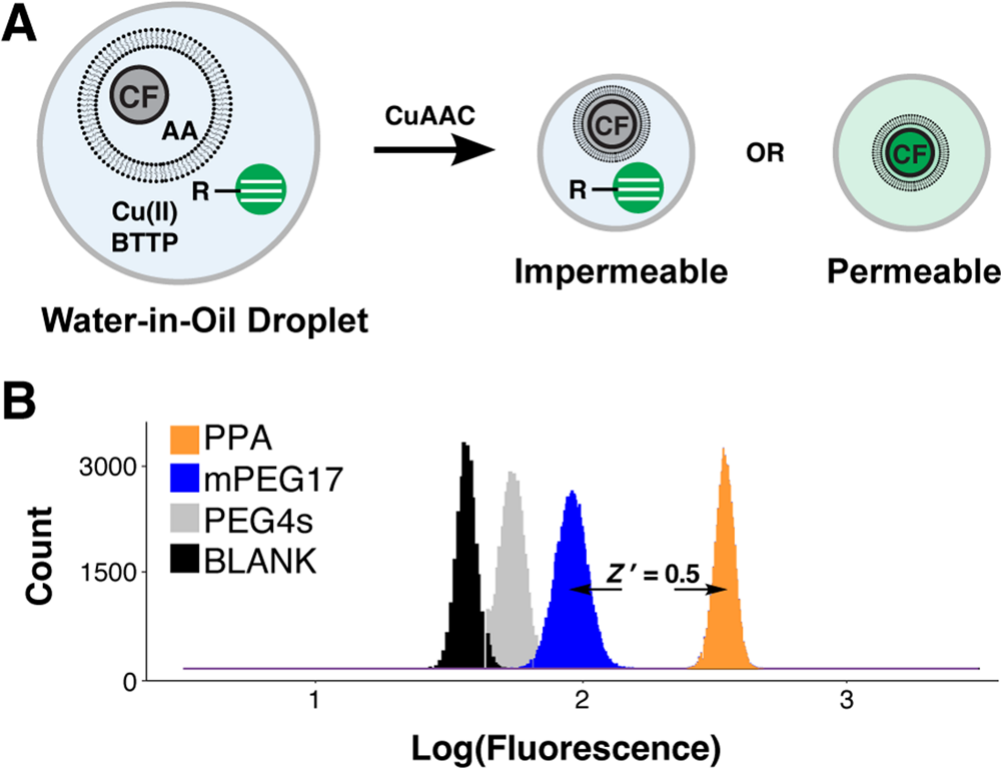
High-throughput microfluidic permeation measurement schematic and droplet assay development. (A) Liposomal CF-N_3_ and alkyne were mixed and encapsulated in microfluidic droplets with Cu(II) and ligand. Fluorescence increase with CuAAC reaction progress transduced compound permeation. (B) PPA, mPEG17, PEG4s, or buffer blank was loaded into droplets together with liposomal CF-N_3_. Droplets were incubated (70 min), and CuAAC reaction progress was detected using laser-induced fluorescence. The assay quality score, *Z*′, was calculated using PPA and mPEG17 as permeation positive and negative control compounds, respectively.

The robust performance of the liposomal permeation assay in microplate format suggested that it could be useful for DEL screening if miniaturization to microfluidic droplet scale was feasible. Conceptually, the liposomal CF-N_3_ probe would be loaded into microfluidic water-in-oil droplets together with alkyne-labeled molecule; CuAAC reagents would be present either in the droplet (copper, ligand) or with the probe in the SUVs (ascorbic acid reducing agent). Membrane-permeable alkynes would transit the membrane and then react with the probe, generating fluorescent triazole product and increasing droplet fluorescence while membrane-impermeable alkynes would not (Figure 3A). Assay performance was quantified in flow injection analysis, wherein droplets are formed by combining control alkynes (25 μM, either PPA positive control or mPEG17 negative control) with SUVs and incubated online (70 min).^45^ Droplet fluorescence was then detected for each injected sample, and droplet fluorescence populations were fitted to normal distributions to calculate the microfluidic *Z*′ = 0.5 using PPA and mPEG17 (Figure 3B). Assay optimization included choice of Cu(I) ligand, online incubation time, alkyne concentration dependence, and compatibility with density additive dextran (SI, Figures S4, S6, and S7). The optimal configuration for microfluidic permeation screening was BTTP ligand, 9% w/v dextran, 25 μM alkyne, and 70 min online incubation.

Flow injection analysis confirmed the compatibility of the liposomal permeation assay with microfluidic droplet-scale screening. It was not immediately obvious that the assay would be droplet-compatible. First, the lipids used for SUV formation are surfactants and might have destabilized the microfluidic emulsion, but we did not observe this behavior in perfluorinated oil.^39^ Second, more hydrophobic CuAAC ligands might have undesirably partitioned into the oil, compromising reaction kinetics. We used advanced Cu(I) ligands with enhanced water solubility to disfavor oil partitioning.^46^ However, this was a nuanced optimization, as THPTA exhibited superior CuAAC performance in free solution, but not in the liposomal assay, presumably due to its low predicted membrane permeability (cLogP; SI, Figure S4). Third, it was not clear whether density additives required for droplet-scale screening^47^ would negatively impact assay performance; assay performance remained robust even with dextran (SI, Figures S6 and S7). In spite of these concerns, optimization yielded a microfluidic assay that was ready for proof-of-concept bead screening.

### Model OBOC Permeability Screening

**Figure 4:**
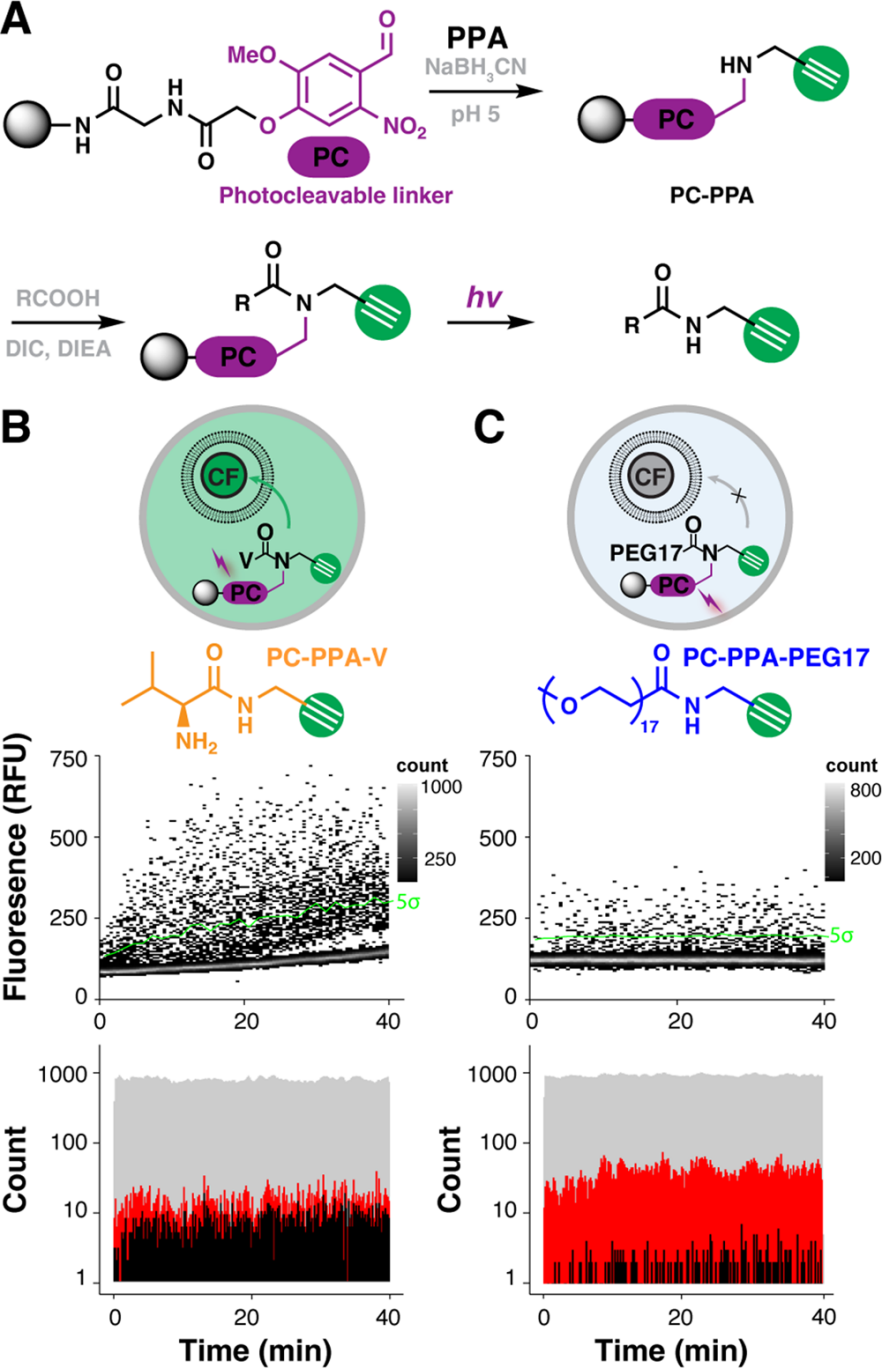
Alkyne-tagging photolinker and permeation assay in microfluidic droplets. (A) Reductive amination of aldehyde photocleavable (PC) linker with PPA yielded the alkyne-tagging photolinker (PC–PPA). Subsequent acylation with various acids yielded photocleavable alkyne-tagged permeation control beads (PC–PPA–V/PC–PPA–PEG17). Alkynes were released from the bead for permeation assay upon UV irradiation. Control beads (B) PC–PPA–V and (C) PC–PPA–PEG17 were evaluated in droplet-scale liposomal permeation assays. The photocleaved product, PPA–V, is a permeable positive control that increases fluorescence of the liposomal probe; PPA–PEG17 an impermeable negative control that does not increase fluorescence of the liposomal probe. Transient heat maps display the ouput from microfluidic droplet-scale permeation assays of PC–PPA–V and PC–PEG17 control beads. A calculated threshold 5 standard deviations (5σ) above the mean negative signal (green line) was used to calculate the false discovery rate. The droplet generation rate (gray), bead occupancy rate (red), and hit rate (black) were binned for the PC–PPA–V and PC–PEG17 microfluidic analyses in 30 s windows.

We next sought to demonstrate that all steps involved in microfluidic droplet-scale functional OBOC screening were also feasible. To label all library members with a minimal N-propargylamide tag, we designed and synthesized an alkyne-tagging photolinker (Figure 4A). Solid-phase synthesis resin was functionalized with an aldehyde-activated photocleavable linker and coupled with propargylamine to yield photocleavable propargylamine (PC–PPA), which could be subsequently acylated with various control carboxylic acids (PEG17, valine) to yield photocleavable permeation control alkyne tool beads (PC–PPA–PEG17, PC–PPA–V; SI,Figure S8). Photocleavage products were characterized by mass spectrometry and by fluorogenic CuAAC reaction monitoring (SI, Table S2 and Figures S9–S14). Assay quality score *Z*′ = 0.7 was measured between fluorescence gain folds of PC–PPA–V and PC–PPA–PEG17 in liposomal permeation plate assays (SI, Figure S9B). The two different control bead types (PC–PPA–PEG17, PC–PPA–V) were separately loaded into microfluidic liposomal permeation assay droplets for subsequent on-chip UV photocleavage and fluorescence detection.^36,48^ Hit droplets were identified as high fluorescence signal (λ_em_ = 530 nm), such that droplet fluorescence was 5σ greater than the mean fluorescence; droplet generation rate, bead introduction rate, and hit rate were plotted as a function of run time (Figure 4B,C). Permeation signal from positive control PC–PPA–V beads was evaluated with and without UV irradiation; permeation signal was only observed with photocleavage (SI, Figure S15).

Photocleavable control bead signal in the droplet liposomal permeation assay agreed with the previously measured values in microplates and further confirmed compatibility of all on-chip OBOC analysis steps. We had previously shown that valine-*N*-propargylamide was permeable,^38^ and we again observed that behavior in these droplet assays. Impermeable PEG17 cleaved from resin yielded marginal permeation signal. Given the 5σ sort threshold (*P* = 3 × 10^–7^) and 40 000 droplets measured over 20 min, 0.01 false discoveries would be expected, thus the source of signal from the PC–PPA–PEG17 beads derives from other systematic sources. Beads traversing the laser focus generate a ”bead spike,” which is useful for approximating droplet occupancy.^37^ Although these spikes are smoothed, larger spikes (from multibead droplets) are not smoothed, resulting in false positive signals. Multibead droplets also inherently deliver high [alkyne], and because transport is concentration dependent, this is another mechanism for generating false positive signals. We have previously shown that multibead droplets are part of a larger Poisson distribution-derived source of error in OBOC DEL screening; identifying replicate hits in screens of multiple library equivalents completely mitigates these sources of error.^36^

The validated fluorogenic CuAAC liposomal permeation assay developed here introduces important throughput and generality advantages that will be useful for library screening. Known membrane-permeable and -impermeable molecules via gold-standard PAMPA assays behaved similarly in the liposomal permeation assay, as did cell-permeable macrocyclic peptides. The assay was compatible with a broad range of lipids, requiring only a minimal alkyne tag that can be readily introduced during solid-phase library synthesis. Finally, the assay is homogeneous, fluorescence-based and compatible with droplet-scale assays, suggesting that it could be implemented for high-throughput solid-phase DEL screening.^37^

The assay’s high-throughput screening compatibility with microplates or droplets is particularly important. The transwell format and LC-MS detection of PAMPA and MDCK assays make implementation in high-throughput settings difficult, thus limiting the scope of structure–permeation relationship measurements. Significant technology development efforts have aimed at transforming these assays into homogeneous fluorescence-based approaches^14,19^ or implementing liposomal formulations.^39^ A recent pooled library screening approach demonstrated the deep structure–permeation insights that can be gained, such as how stereochemistry and *N*-methylation affect macrocycle permeability.^32^ The present technology offers opportunities to build on these capabilities by screening individual library members and pairing with nucleic acid encoding, a highly parallel and generalized structure deconvolution strategy to increase the breadth of structure–permeation measurements.^49^ Combinatorial synthesis in particular would allow ready access to bRo5 space, where permeation trends remain largely unclear.^7,50−52^

Future studies will seek to draw direct correlations between the formal permeation coefficient and the PC50 and establish the generality of the alkyne labeling strategy. While the liposomal assay does not readily yield defined quantitative permeation metrics, such as the permeation coefficient, *P*_e_, the PC50 of the concentration-dependent permeation curve makes inroads toward establishing such a metric. The liposomal membrane format does offer a potentially important advantage, however, in being a unilamellar membrane bilayer. A recent study showed that the PAMPA membrane thickness is dramatically higher than that of a bilayer and may not accurately approximate passive diffusion through a cellular bilayer.^23^ Further evaluation of this assay in library contexts may shed light on the importance of a more accurate cellular membrane model during permeation screening. Finally, screening libraries of model alkynes, obtained either through site-selective labeling (a staple of modern chemical biology workflows) or installed as a linker during combinatorial synthesis as described above, could generate the requisite correlations. Furthermore, dose–response microfluidic screening by modulating photocleavage intensity^48^ could provide such a link at library scale.

Moving toward library screening, there are several minor considerations. It may be necessary to identify and minimize false positive signals given a potentially high ”permeation hit” rate. False positive permeation hits are most likely to be synthesis truncates or membranolytic. Synthesis truncates are potentially false positives because they are likely to be more membrane-permeable on the basis of lower molecular weight and fewer hydrogen bond donors/acceptors. Truncate artifacts are a known and accepted possibility in combinatorial library screening, and emerging machine learning-based synthesis prediction strategies could identify building blocks or building block pairs with poor coupling efficiency, resulting in truncation.^53^ Library members that induce membrane lysis will also register as permeation hits but are uninteresting from the perspective of transport. Inclusion of an orthogonal liposomal dequenching assay using a dye with different emission properties could allow us to discriminate lysis from passive diffusion.^39^ The resulting multiplexed assays could advantageously offer the possibility of discovering structures that disrupt membranes, a common property of antimicrobial peptides and candidates for drug delivery through endosome escape.^39,54^

## Conclusions

In conclusion, we have developed and validated a fluorogenic CuAAC-based liposomal permeation assay that is compatible with high-throughput screening applications. The assay sensitively measures the permeation of known control molecules and structurally novel bRo5 macrocyclic peptides. These bRo5 molecules hold great promise to ligand difficult targets, but structure–permeation relationships that could guide the development of these molecules into clinical assets must be established. The liposomal permeation assay of this study paves the way to bRo5 DEL design, synthesis, and screening experiments that will allow us to generate the needed empirical data and build models that will ultimately reveal such relationships.

## Experimental Section

### Materials

All reagents were purchased from MilliporeSigma (St. Louis, MO) unless otherwise specified. Tris[(1-benzyl-1*H*-1,2,3-triazol-4-yl)methyl]amine (TBTA, >95% HPLC, 1Click Chemistry, Tinton Falls, NJ), BTTP (>95% HPLC, Click Chemistry Tools, LLC, Scottsdale, AZ), 5(6)-carboxytetramethylrhodamine (cTMR, Anaspec, Fremont, CA), CalFluor 488 azide (CF-N_3_, Click Chemistry Tools, LLC, Scottsdale, AZ), l-ascorbic acid (Acros Organics, Thermo Fisher Scientific, Inc., Waltham, MA), 1,2-dioleoyl-*sn*-glycero-3- phosphocholine (DOPC, 10 mg/mL in chloroform, Avanti Polar Lipids, Alabaster, AL), 1-palmitoyl-2-oleoyl-*sn*-glycero-3-phosphocholine (POPC, 10 mg/mL in chloroform, Avanti Polar Lipids), Brain Polar Lipid Extract (Porcine, 25 mg/mL in chloroform, Avanti Polar Lipids), *E. coli* Polar Lipid Extract (25 mg/mL in chloroform, Avanti Polar Lipids), 1,2-dioleoyl-*sn*-glycero-3-phospho-(1′-*rac*-glycerol) (sodium salt) (DOPG, 25 mg, Avanti Polar Lipids), cholesterol (Ch, Ark Pharm, Inc., Arlington Heights), m-PEG17 acid (BroadPharm, San Diego, CA), m-PEG4-propargyl (mPEG4, BroadPharm, San Diego, CA), m-PEG8-propargyl (mPEG8, BroadPharm, San Diego, CA), m-PEG17-propargyl (mPEG17, BroadPharm, San Diego, CA), propargyl-PEG4-sulfonic acid (PEG4s, BroadPharm, San Diego, CA), Slide-A-Lyzer dialysis cassettes (10K MWCO, 3 mL, Thermo Fisher Scientific), dextran from Leuconostoc spp. (*M*_*r*_: ∼70 000, Dextran 70, MilliporeSigma, St. Louis, MO), PAMPA multiscreen Transport (up) (Merck Millipore: cat, MAIPN4550; lot, R4PA35207), Multiscreen Transport (bottom) (Merck Millipore: cat, MATRNPS50; lot:, 623915), 3 M Novec 7500 Engineered Fluid (3M, Saint Paul, MN), and Pico-Surf (5% (w/w) in Novec 7500, Sphere Fluidics, Cambridge, UK) were used as provided. All synthesized compounds had purity >95% by LC-MS analysis.

### Buffers

PBS buffer (0.01 M phosphate buffer, 2.7 mM KCl, and 137 mM NaCl, pH 7.4), PBS wash buffer (0.01 M phosphate buffer, 2.7 mM KCl, and 137 mM NaCl, 0.04% Tween-20, pH 7.4), and Bis-Tris propane wash buffer (BTPWB, 50 mM NaCl, 0.04% Tween-20, 10 mM Bis-Tris, pH 7.6) were prepared in DI H_2_O.

### Parallel Artificial Membrane Permeability Assay (PAMPA)

A modified PAMPA was used for compound permeability measurements and was performed by Pharmaron. Briefly, alkyne stock solution (1 mM in DMSO) was diluted (50 μM final in PBS, pH 7.4). The lipid solution (1.8% w/v egg lecithin in dodecane, 5 μL spotting volume) was added to each acceptor well of the multiscreen-IP filter plate. PBS (300 μL) was added to all wells of the acceptor plate, and diluted alkyne stock (300 μL) was added to the wells of the donor plate in triplicate. The plate was assembled and incubated (16 h, 37 °C). An aliquot of donor well sample (2.5 μL) was diluted in PBS (47.5 μL), and an aliquot of the acceptor well (50 μL) was transferred to a 96-well analysis plate. Internal standard (100 nM alprazolam, 200 nM caffeine, 200 nM diclofenac in 100% MeOH) was added to all samples. Samples were vortexed and centrifuged (20 min, 3220*g*), then analyzed by LC-MS/MS.

The effective permeability (*P*_e_) was calculated as
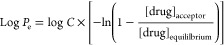
1
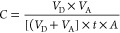
2where *V*_D_ is the donor compartment volume (0.3 mL), *V*_A_ is the acceptor compartment volume (0.3 mL), *A* is the filter area (0.24 cm^2^), and *t* is the time (16 h).

### Liposome Generation and Characterization

DOPC or DOPG (10 mg/mL, 500 μL) were pipetted into a scintillation vial (20 mL), or DOPC (10 mg/mL, 400 μL) and POPC (10 mg/mL, 100 μL) were pipetted into a scintillation vial (20 mL). Samples were dried under argon then evaporatively dried in a vacuum desiccator (16 h). Degassed PBS buffer was combined with CF-N_3_ probe (1, 4, or 20 μM final) and ascorbic acid (AA, 10 mM). Lipid film was resuspended using degassed probe/AA/PBS buffer solution (1 mL), and the vial was incubated on a hot plate (15 min, 37 °C) with gentle vortexing (every 5 min). Lipid suspension was sonicated (1 min, Branson 3510 DTH ultrasonic cleaner) followed by rest (1 min), repeating 9 additional times. Liposomes were generated by extrusion (11 passages, 100 nm polycarbonate membrane, Mini-Extruder, Avanti Polar Lipids). Liposome solution was loaded into dialysis cassettes (10K MWCO, 3 mL Slide-A-Lyzer) and incubated in PBS buffer (1 L, 16 h, 4 °C). Liposome size distributions and stability were analyzed by dynamic light scattering (DLS, Zetasizer Nano ZS, Malvern Panalytical, United Kingdom).

The same protocol was followed to produce and characterize liposomes with other lipid compositions. DOPC (10 mg/mL, 400 μL), POPC (10 mg/mL, 100 μL), and cholesterol (5 mg/mL, 50/100 μL for 10%/20% mole of cholesterol, respectively) were combined, dried, and rehydrated with probe solution in scintillation vials. Brain polar lipid extract (25 mg/mL, 200 μL) or *E. coli* polar lipid extract (25 mg/mL, 200 μL) were dried and rehydrated with probe solution in scintillation vials.

### Microplate-Scale Permeation Assay

CuSO_4_ (4 mg) was dissolved in DI H_2_O (500 μL, 50 mM), l-ascorbic acid (8.8 mg) was dissolved in PBS (500 μL, 100 mM), TBTA (2.7 mg) was dissolved in DMSO (509 μL, 10 mM), and mPEG17/mPEG8/mPEG4/PEG4s/propargyl amine/Cyc1–4 stock solutions (50 mM, with the purity > 95% by HPLC analysis) were prepared in DMSO, and CF-N_3_ was dissolved in DMSO (2 mM). Fluorogenic CuAAC reaction premixture was prepared by combining CuSO_4_ (10 μL) and TBTA (8 μL) in PBS (982 μL). Alkyne analytes were diluted (0.1 mM in CuAAC reaction premixture +2% DMSO). Free probe solution was prepared with CF-N_3_ (4 μM) and l-ascorbic acid (10 mM) in PBS buffer. Alkyne analyte in CuAAC premixture (5 μL) or CuAAC premixture +2% DMSO (5 μL) blank sample was combined with liposome (5 μL) or free probe solution (5 μL) in microtiter plate wells (384-well, Greiner, Thermo Scientific). Transient assay fluorescence (λ_ex_/λ_em_ = 488/530 nm) was detected using a multimode plate reader (CLARIOstar, BMG). Fluorescence fold gain was calculated by dividing the sample fluorescence by blank fluorescence. Initial velocities (*V*_0_) were obtained by linear regression analysis of the fluorescence fold gain after the initial lag period.

### Microplate-Scale Concentration-Dependent Permeation (PC50)

Assays were assembles as described above, combining liposomal probe (5 μL; 4 μ M CF-N_3_, 10 mM ascorbic acid, *E. coli* lipid SUV) with alkyne sample (5 μL) but with varying concentrations of mPEG4, mPEG8, mPEG17, and PEG4s (0.0001/0.001/0.005/0.01/0.05/0.1/0.5/1/5 mM) or Cyc1–4 (0.0001/0.001/0.005/0.01/0.05/0.1/0.5 mM). Broken liposomal *V*_0_ was obtained by combining liposomal probe (5 μL) with Triton X-100 solution (0.42 μL, 10% w/v), then adding the highest concentration sample for each alkyne (5 μL; 0.5 mM Cyc1–4 or 5 mM PEG samples). Transient assay fluorescence (λ_ex_/λ_em_ = 488/530 nm) was detected using a multimode plate reader (CLARIOstar). Each [alkyne] *V*_0_ was divided by the corresponding broken liposome maximum [alkyne] *V*_0,broken_. These normalized data were fit to a sigmoid to determine the [alkyne] at which *V*_0_/*V*_0,broken_ was 50% maximum (PC50).

### Droplet-Scale Permeation Measurement

Microfluidic droplet-based screening was performed in a UV-free room as previously described.^37,47^ Briefly, perfluorous oil phase (Novec 7500) with stabilizer (4% Pico-Surf) was loaded into a syringe (1 mL, BD Tuberculin, BD Medical, Franklin Lakes, NJ) fitted with blunt-end needle (30 gauge QuantX, Fisnar, Germantown, WI) and connected to the microfluidic device OIL1 inlet via Tygon microbore tubing (0.01” I.D. × 0.03” O.D. × I.D., United States Plastic Corp.). Aqueous inputs (AQ1 and AQ2, see below) were loaded into the syringes and connected to AQ inlets of the microfluidic device. Novec 7500 was loaded into syringes (10 mL) and similarly connected to the device via Tygon microbore tubing as spacing oil (OIL2) and flow focusing oil (OIL3). The incubation channel was primed with Novec 7500 by filling from the OIL2 and OIL3 inputs before initiating flows from OIL1 and AQ inlets. OIL and AQ flows were driven by syringe pumps (Legato 101, KD Scientific, Holliston, MA): AQ1 (400 nL/min) AQ2 (400 nL/min), OIL1 (500 nL/min), OIL2 (16 μ L/min), and OIL3 (6 μL/min). Droplets were equilibrated (15/70 min) prior to beginning data acquisition.

AQ1 was prepared by combining *E. coli* polar lipid SUV suspension (488.75 μL, 20 μM CF-N_3_ + 10 mM ascorbic acid), CuSO_4_ (5 μL, 50 mM in DI H_2_O), and BTTP (6.25 μL, 20 mM in DMSO). For flow injection analysis experiments, AQ2 included alkyne (PPA, mPEG17, or PEG4s; 1 mM, 0.1 mM, or 0.025 mM) and cTMR (0.2 μM) with or without dextran (9%, w/v) in PBS. For bead analysis experiments, AQ2 included (PC–PPA-V/PC–PPA–mPEG17, 600 beads/μL, see Supporting Information) with cTMR (0.2 μM) and dextran (9% w/v) in PBS buffer. After beads were encapsulated, they were irradiated in flow using a custom optical fiber patch cable (600 μm diameter, 0.39 NA, Thorlabs) that was coupled to a high-power UV LED (365 nm, Thorlabs).^48^ All experiments used the maximum LED voltage (5 V).

Fluorescence data were acquired using a custom confocal laser-induced fluorescence detection system. Droplets were detected based on internal standard (cTMR) signal in PMT 2 as previously described.^36^ CF-N_3_ probe fluorescence was detected in PMT 1. PMT data were digitized using a DAQ board (12 kHz, NI-USB-6341, National Instruments, Austin, TX) and processed using custom data acquisition control software written in LabVIEW (National Instruments).^36−38^ Median smoothing (window width = 5) was applied to both channels. Once a droplet was detected, the average PMT 1 signal was calculated. Data analysis for droplet-scale *Z*′ determination, droplet generation, bead occupancy, and hit rate visualization was performed as previously described.^37^

## Supporting Information Available

Additional experimental methods, results and compound characterization data (PDF)

## Abbreviations Used

SUV: small unilamellar vesicle
DOPC: 1,2-dioleoyl-*sn*-glycero-3-phosphocholine
POPC: 1-palmitoyl-2-oleoyl-*sn*-glycero-3-phosphocholine
DOPG: 1,2-dioleoyl-*sn*-glycero-3-phospho-(1′-*rac*-glycerol)
Ch: cholesterol
*P*_*e*_: effective permeability values
cLogP: calculated partition coefficient
TBTA: tris[(1-benzyl-1*H*-1,2,3-triazol-4-yl)methyl]amine
AA: ascorbic acid
PPA: propargylamine
CF-N_3_: CalFluor 488 azide
PEG: polyethylene glycol
PAMPA: parallel artificial membrane permeability assay
CuAAC: copper-catalyzed azide alkyne cycloaddition
PC: photocleavable
Ro5: the Rule of 5
bRo5: beyond the Rule of 5
DEL: DNA-encoded library
OBOC: one-bead-one-compound
