# Supplementary material for: Liposomal Permeation Assay for Droplet-Scale Pharmacokinetic Screening
Source: J Med Chem. 2023 Apr 19;66(9):6288–96. doi: 10.1021/acs.jmedchem.3c00138 (PMC10184116; doi:10.1021/acs.jmedchem.3c00138)
Supplement: Supplementary file 1 — jm3c00138_si_001.pdf [file jm3c00138_si_001.pdf]

Supporting Information: Liposomal Permeation Assay for Droplet-Scale Pharmacokinetic Screening

Juan Hu,[†] Alix I Chan,[‡] Emel Adaligil,[‡] Ivy Kekessie,[‡] Mifune Takahashi,[¶] Aimin Song,[‡] Christian N. Cunningham,[‡] and Brian M. Paegel[*,†,§]

†*Department of Pharmaceutical Sciences, University of California, Irvine*

‡*Department of Peptide Therapeutics, Genentech, South San Francisco, California 94080*

¶*Department of Drug Metabolism and Pharmacokinetics, Genentech, South San Francisco, California 94080*

§*Departments of Chemistry & Biomedical Engineering, University of California, Irvine*

E-mail: bpaegel@uci.edu

Contents

Materials **S-4**

Synthesis of alkyne-labeled macrocycles **S-4**

Synthesis of alkyne-labeled cell-penetrating peptides DD5o I/II/III **S-6**

Synthesis of QC beads **S-7**

Synthesis of PC-PPA linker and control beads **S-8**

Control bead preparation **S-9**

Bulk resin photocleavage **S-10**

References **S-11**

Supplemental Figures **S-12**

Figure S1. Liposome characterization via dynamic light scattering (DLS) S-13

Figure S2. Structures of macrocyclic peptides with and without alkyne labeling . S-14

Table S1. Permeation coefficients of controls, alkyne-tagged macrocycles and their parent molecules S-15

Figure S3. Liposomal permeation assay using different lipid mixtures S-16

Figure S4. Ligand test for liposomal permeation assay S-16

Figure S5. Cell-penetrating peptide design, synthesis, and assay S-16

Figure S6. Density additive effect on liposomal permeation assay S-19

Figure S7. Microfluidic permeation flow injection analysis S-19

Figure S8. Solid-phase synthesis of PC-PPA-V and PC-PPA-PEG17 tool compound beads S-21

Table S2. MALDI-TOF MS analysis of microcleavage products from QC resins . . S-21

Figure S9. Liposomal permeation assay of photocleaved control compounds . . . S-24

Figure S10. MALDI-TOF MS analysis of K(Mca)-R S-24
Figure S11. MALDI-TOF MS analysis of PC4 linker S-25
Figure S12. MALDI-TOF MS analysis of PC-PPA S-26
Figure S13. MALDI-TOF MS analysis of PC-PPA-PEG17 . . . . S-27
Figure S14. MALDI-TOF MS analysis of PC-PPA-V S-28
Figure S15. Microfluidic photodosing control experiment S-29
Figure S16. LC-MS analysis of macrocyclic peptide Cyc1 . . . . S-30
Figure S17. LC-MS analysis of macrocyclic peptide Cyc2 . . . . S-31
Figure S18. LC-MS analysis of macrocyclic peptide Cyc3 . . . . S-32
Figure S19. LC-MS analysis of macrocyclic peptide Cyc4 . . . . S-33
Figure S20. Characterization of DD5o I/II/III peptides S-33

Materials

All reagents were purchased from MilliporeSigma (St. Louis, MO) unless otherwise specified. 1,3-Bis[tris(hydroxymethyl)methylamino]propane (Bis-Tris), trifluoroacetic acid (TFA), triisopropylsilane (TIPS), tris(2-carboxyethyl)phosphine (TCEP), α-cyano-4-hydroxycinnamic acid (HCCA) (Life Technologies, Carlsbad, CA), N,N′-diisopropylcarbodiimide (DIC, Acrõs Organics, Fair Lawn, NJ), N_α-Fmoc-N_ω-(2,2,4,6,7-pentamethyldihydrobenzofuran-5-sulfonyl)-L-arginine (Fmoc-R(Pbf)-OH, Thermo Fisher Scientific). N,N-dimethylformamide (DMF, Thermo Fisher Scientific, Waltham, MA), Dimethyl sulfoxide (DMSO, AMRESCO Inc., Solon, OH), N-α-Fmoc-N-ε-7-methoxycoumarin-4-acetyl-lysine (Fmoc-Lys(Mca)-OH, EMD Millipore, Billerica, MA), N-α-Fmoc-glycine (Fmoc-Gly-OH, AnaSpec, Inc., Fremont, CA), N-Fmoc-L-propargylglycine (Fmoc-Pra-OH, Combi-Blocks, San Diego, CA), N-Fmoc-3-cyclohexyl-L-alanine (Fmoc-Cha-OH, Chem-Impex, Wood Dale, IL), N-Fmoc-N-methyl-L-alanine (Fmoc-MeA-OH, Chem-Impex), N-Fmoc-N-hexyl-glycine (Fmoc-HxG-OH, Acrotein, Hoover, AL), N-Fmoc-4-chloro-L-phenylalanine (Fmoc-Phe(4-Cl)-OH, Chem-Impex), N-Fmoc-β-tert-butyl-L-alanine (Fmoc-tBuA-OH, AnaSpec), N-Fmoc-N-methyl-L-leucine (Fmoc-MeL-OH, Matrix Scientific, Columbia, SC), N-Fmoc-L-norleucine (Fmoc-Nle-OH, Chem-Impex), N-Fmoc-N-ethyl-glycine (Fmoc-EtG-OH, Acrotein), and 2-(4-formyl-2-methoxy-5-nitrophenoxy)acetic acid (PC-CHO, Enamine, Monmouth Jct., NJ), and 1,2-Bis(bromomethyl)benzene (97%, Frontier Scientific Inc., Newark, DE) were used as provided. All compounds had purity $>$ 95% by HPLC.

Synthesis of alkyne-labeled macrocycles

Methyl indole AM resin (2 g, loading 0.69 mmol/g, 1.38 mmol) was swollen in DMF (40 mL) at room temperature for 20 min. The supernatant was removed. A 20% solution of 4-methylpiperidine in DMF (40 mL) was added to the resin. The resulting mixture was ag-

itated at room temperature for 10 min. The supernatant was drained, and the process was repeated once. The resin was then washed thoroughly with DMF (40 mL × 6). A solution of Fmoc-Pra-OH (1.39 g, 4.14 mmol, 3.0 equiv) and 2-(1H-7-azabenzotriazol-1-yl)-1,1,3,3-tetramethyl uronium hexafluorophosphate (HATU, 1.57 g, 4.13 mmol, 3.0 equiv) in DMF (20 mL) was added to the resin, followed by the addition of N,N-diisopropylethylamine (DIEA, 0.721 mL, 4.14 mmol, 3.0 equiv). The resulting mixture was agitated at room temperature overnight. Ninhydrin test indicated completion of reaction. The supernatant was drained. The resin was washed with DMF (40 mL × 3) and DCM (40 mL × 3), and was dried in vacuo.

The linear precursors of macrocyclic peptides Cyc1-4 were synthesized using automated microwave-assisted solid-phase peptide synthesis on a Liberty Blue™ Peptide Synthesizer (CEM). The methyl indole AM resin preloaded with Fmoc-Pra-OH was used as the solid support, and DIC/OxymaPure® as the activating system. In the coupling steps, a solution of an Fmoc-protected amino acid (0.4 M, 5 mL) in DMF was added to the resin (0.30 g, 0.21 mmol), followed by the addition of OxymaPure® solution (1 M, 2 mL) and DIC solution (1 M, 2 mL) in DMF. The resulting mixture was purged with nitrogen under microwave irradiation at 90 °C for 2 min with the exceptions of cysteine (75 °C, 2 min). The supernatant was drained, and the process was repeated once. The supernatant was drained, and the resin was washed with DMF (10 mL × 3). For Fmoc removal, a 20% solution of 4-methylpiperidine in DMF (10 mL) was added to the resin. The resulting mixture was purged with nitrogen under microwave irradiation at 90 °C for 1 min. The supernatant was drained, and the process was repeated once. The resin was then washed thoroughly with DMF (10 mL × 5). The coupling-deprotection cycle was repeated until the peptide assembly was complete. The resin was then agitated with a solution of chloroacetic anhydride (0.359 g, 2.1 mmol, 10.0 equiv) and DIEA (0.366 mL, 2.1 mmol, 10.0 equiv) in 10 mL DMF at room temperature overnight. Ninhydrin test indicated complete reaction. The supernatant was drained. The resin was washed with DMF

(10 mL × 3) and DCM (10 mL × 3), and was dried in vacuo.

The N-chloroacetylated peptides were released from the solid support by TFA treatment. A mixture of TFA/phenol/water/thioanisole/triisopropylsilane (v/w/v/v/v 90 : 2.5 : 1.5 : 3.5 : 2.5, 8 mL) was added to the resin (0.30 g) at 0 °C. The resulting mixture was slowly warmed to room temperature and allowed to mix for 3 h. The supernatant was collected by filtration, and the resin was washed with the cleavage mixture (4 mL × 3). The combined filtrate was concentrated under reduced pressure to a small volume, which was diluted with anhydrous diethyl ether (45 mL). The resulting mixture was allowed to stand at 0 °C for 30 min and then centrifuged. The precipitate was washed with anhydrous diethyl ether (45 mL × 3) and dried in vacuo.

The obtained crude linear peptide was dissolved in 50% acetonitrile (ACN) in water at a concentration of 10 mg/mL, and the pH was adjusted to 8 – 9 by dropwise addition of DIEA. The reaction was stirred overnight and was monitored with liquid chromatography-mass spectrometry (LC-MS). Upon completion of cyclization, the solution was lyophilized to dryness. The obtained crude macrocyclic peptides were purified by reverse-phase high-performance chromatography with purity > 95% (RP-HPLC, C18 column, 21 mm × 250 mm, 5 µm, 100 Å, 10 mL/min, 60-min gradient from 30 to 80 % ACN/water containing 0.1% TFA).

Synthesis of alkyne-labeled cell-penetrating peptides DD5o I/II/III

DD5o-I/II/III cell-penentrating peptides were prepared via standard solid-phase synthesis using Rink Amide resin (100-200 mesh, 0.6 mmol/g, 100 mg) and Fmoc-protected amino acid building blocks. Resin was loaded into a fritted syringe (5 mL, Torviq). For each coupling, Fmoc amino acid (0.6 mmol, 10 equiv), OxymaPure® (0.6 mmol, 10 equiv), DIC (0.6 mmol, 10 equiv), and DIEA (1.2 mmol, 20 equiv)) were dissolved in DMF (2 mL).

The solution was combined with resin and incubated with rotation (60 min, 50 °C, 8 rpm) and then expelled. The resin was washed (3 × DMF, 3 × DCM, 3 × DMF), then Fmoc was removed (20% piperidine in DMF, 1 × 5 min, 1 × 15 min, RT, 8 rpm).

Cleavage cocktail (90% TFA, 5% DCM, 5% TIPS, 200 µL) was added to dried resin and the reaction was incubated (1 h, RT). The cleavage solution was evaporated to dryness in a rotary evaporator and the pellet was washed with cold diethyl ether. The pellet was dried and dissolved in 1:1 ACN : 20 mM ammonium bicarbonate (1 mM final peptide concentration), and the pH was adjusted to 8.0 using NaOH (1 M in DI water).[1] Bis(bromomethyl) linker (1,2-Bis(bromomethyl)benzene) was added (1.5 equiv, 1.5 mM in the final reaction solution) and the peptide and incubated with rotation (1 h, RT, 8 rpm). Cyclized peptides were HPLC purified (column: XBridgeTM Prep C18 5 μm 10x150 mm column; solvent A: 0.1%TFA in H_2O, solvent B: MeOH; B varied from 30 to 90% in 25 min; flow rate: 3.5 mL/min) and characterized by MALDI-TOF MS as previously described (Fig. S20).

Synthesis of QC beads

All resin synthesis, bead handling, and microfluidic droplet-based screening were performed in a UV-free room as previously described. Linker synthesis proceeded via iterative cycles of solid-phase synthesis. All spin-column wash and reaction volumes were identical (0.4 mL) unless noted. All fritted syringe wash and reaction volumes were in accordance with the fritted syringe volume unless noted.

Synthesis resin (TentaGel® MB RAM, size: 200 - 250 µm dia., capacity: 0.2 - 0.3 mmol/g, 200 mg, Rapp-Polymere) was transferred to a separate fritted spin-column (Mobicol Filters (Large/Upper), 10µm Pore Size, Boca Scientific Inc, Dedham, MA), washed (3 × DCM, 3 × DMF), and swelled in DMF (16 h, RT). Subsequent amino acid coupling cycles of Fmoc-Lys(Mca)-OH and Fmoc-R(Pbf)-OH consisted of: (1) Fmoc removal (20% piperidine in DMF, 1 × 5 min, 1 × 15 min, 8 rpm, RT) and washing (3 × DMF, 3 × DCM,

3 × DMF); (2) N-α-Fmoc-amino acid (120 μmol) activation with COMU/DIEA (120/240 μmol, 2 min, RT); (3) addition of activated N-α-Fmoc-amino acid to resin and incubation (1 h , 50 °C, 8 rpm); (4) capping (20% acetic anhydride in DMF, 30 min, 50 °C, 8 rpm) and washing (6 × DMF, 3 × DCM, 3 × DMF).

Resin cleavage and MALDI-TOF MS analysis was performed as previously described.[2] Briefly, QC beads (200 μm) were removed (20 μL) and dried in vacuo. Cleavage cocktail (90% TFA, 5% DCM, 5% TIPS, 200 μL) was added to dried single QC beads, incubated (15 min, RT), and dried in vacuo (60 °C). Compound was resuspended (50% ACN, 0.1% TFA in H_2O, 30 μL) and a diluted (1:10) aliquot (1 μL) was co-spotted onto a MALDI-TOF MS target plate with HCCA matrix solution, dried, and analyzed via MALDI-TOF MS (Microflex, Bruker Daltonics, Inc., Billerica, MA).

Synthesis of PC-PPA linker and control beads

Screening resin (amino-functionalized, 10 μm dia., 0.28 mmol/g, 50 mg, Rapp-Polymere) was loaded into a separate fritted syringe (ISOLUTE® Frits, 6 mL, 13 mm, 10 μm PE, Biotage, Uppsala, Sweden), washed (3 × DCM, 3 × DMF), and swelled in DMF (1 h, RT) before mixing with QC resin (10 mg). Subsequent amino acid coupling cycles of Fmoc-Gly-OH, Fmoc-Pra-OH, and Fmoc-Gly-OH consisted of: (1) Fmoc removal (20% piperidine in DMF, 1 × 5 min, 1 × 15 min, 8 rpm, RT) and washing (3 × DMF, 3 × DCM, 3 × DMF); (2) N-α-Fmoc-amino acid (160 μmol) activation with DIC/Oxyma/DIEA (160/160/320 μmol, 2 min, RT); (3) addition of activated N-α-Fmoc-amino acid to resin and incubation (1 h , 50 °C, 8 rpm); (4) capping (20% acetic anhydride in DMF, 30 min, 50 °C, 8 rpm) and washing (6 × DMF, 3 × DCM, 3 × DMF). PC-CHO (80 μmol) was activated with DIC/Oxyma/TMP (120/80/80 μmol), added to resin. The resin was incubated (2 h, 50 °C, 8 rpm, 2 ×) and washed (3 × DMF, 3 × DCM, 3 × DMF). Resin was washed (3 × DMF), combined with PPA (1 mL, 1 M in 1% acetic acid/DMF v/v), incubated (30 min, 50 °C, 8 rpm), combined

with $NaBH_3CN$ (1 mL, 1 M in 1% acetic acid/MeOH v/v), incubated (30 min, 50 °C, 8 rpm), combined with $NaBH_3CN$ (1 mL, 3 M in 1% acetic acid/MeOH v/v), incubated (1 h, 50 °C, 8 rpm), combined with $NaBH_3CN$ (1 mL, 3 M in 1% acetic acid/MeOH v/v), incubated (1 h, 50 °C, 8 rpm), and washed (3 × DMF, 3 × H_2O, 3 × DMF, 3 × DCM, 3 × DMF). Synthesis of PC-PPA linker was verified by acid cleavage and MALDI-TOF MS analysis (see above).

Dual-scale PC-PPA resin (30 mg) acylation with Fmoc-valine-OH consisted of: (1) Fmoc-valine-OH (80 µmol) activation with DIC/Oxyma/DIEA (80/80/160 µmol, 2 min, RT); (2) addition of activated Fmoc-valine-OH to resin and incubation (1 h, 50 °C, 8 rpm); (3) Fmoc removal (20% piperidine in DMF, 1 × 5 min, 1 × 15 min, 8 rpm, RT) and washing (3 × DMF, 3 × DCM, 3 × DMF). Synthesis of PC-PPA-Val was verified by acid cleavage and MALDI-TOF MS analysis (see above).

Dual-scale PC-PPA resin (30 mg) acylation with m-PEG17-OH consisted of: (1) m-PEG17-OH (80 µmol) activation with DIC/Oxyma/DIEA (80/80/160 µmol, 2 min, RT); (2) addition of activated m-PEG17-OH to resin and incubation (2.5 h, 50 °C, 8 rpm) and washing (3 × DMF, 3 × DCM, 3 × DMF). Synthesis of PC-PPA-PEG17 was verified by acid cleavage and MALDI-TOF MS analysis (see above).

The 200-µm QC resin was removed from both PC-PPA-Val and PC-PPA-PEG17 10-µm screening resin by filtration (CellTrics 150 µm mesh, Sysmex Partec, Lincolnshire, IL). Flow-through was collected, washed (DMF, 10 × 0.5 mL), and resuspended in DMF (0.5 mL).

Control bead preparation

Control beads (PC-PPA-Val or PC-PPA-PEG17) were loaded into a spin column (Mobicol, 10-µm pore, Boca Scientific, Boca Raton, FL), washed with solvent (5 × DMF, 5 × DCM, 5 × MeOH), and air dried (5 min). Beads were resuspended in DMF (0.5 mL), incubated

(1 h, 50 °C, 8 rpm), washed with solvent (5 × DMF, 5 × DCM, 5 × MeOH), and dried as above. This cycle of soaking, washing, and drying the control bead was repeated with three different soaking solvents (DMF, DCM, and PBS wash buffer). The washed beads were stored in DMF (4 °C) until further use.

For each screen, an aliquot of bead in DMF was transferred to an eppendorf tube, pelleted, and exchanged to PBS wash buffer (0.5 mL). The sample was vortexed (1 h), pelleted, and exchanged to fresh PBS wash buffer. The bead aliquot washing procedure (pellet, vortex, exchange to fresh PBS wash buffer) was repeated three times in total. Beads in PBS wash buffer was filtered (CellTrics 20-µm mesh), loaded into a syringe (1 mL, BD Medical, Franklin Lakes, NJ) fitted with a blunt-tip Luer-Lok needle (30-gauge, Small Parts, Inc., Miramar, FL) connected to a microfluidic filter device via Microbore Tygon tubing (0.01 in. i.d. × 0.03 in. o.d., Saint Gobain, Valley Forge, PA), and manually driven through the microfluidic filter.[2] The syringe containing the bead aliquot was refilled with PBS wash buffer (0.5 mL) and the contents were again driven through the microfluidic filter (repeated 3 ×). The filtered bead solution was pelleted and exchanged to fresh PBS wash buffer for quantitation via hemocytometer. Before applied to droplet permeation assay, PC-PPA-V / PC-PPA-PEG17 beads are washed by PBS buffer and switched to AQ2 solution in 1x PBS buffer with cTMR (0.2 µM) and Dextran (9%, w/v) with 600 beads/µL in final, which would be loaded into 1-mL syringe and performed in microfluidic droplet permeation assay.

Bulk resin photocleavage

Prepared resin (1 mg, PC-PPA-V or PC-PPA-PEG17) was washed (3 × MeOH, 200 µL), then suspended in MeOH (200 µL). The tubes were placed in a UV-crosslinking oven (CL-1000L, Analytik Jena, Jena, Germany), exposed with shaking (365 nm, 16 h, 3000 rpm), and centrifuged (100 rcf). The supernatant was removed, dried in vacuo (60 °C),

resuspended in DMSO (5 mL), and further diluted (50 fold) with liposomal permeation assay pre-mixture.

References

(1) Peraro, L.; Siegert, T.; Kritzer, J. Chapter Fourteen Conformational Restriction of Peptides Using Dithiol Bis-Alkylation. *Meth. Enzymol.* **2016**, *580*, 303–332.

(2) Cochrane, W. G.; Malone, M. L.; Dang, V. Q.; Cavett, V.; Satz, A. L.; Paegel, B. M. Activity-Based DNA-Encoded Library Screening. *ACS Combi. Sci.* **2019**, *21*, 425–435.

Supplemental Figures

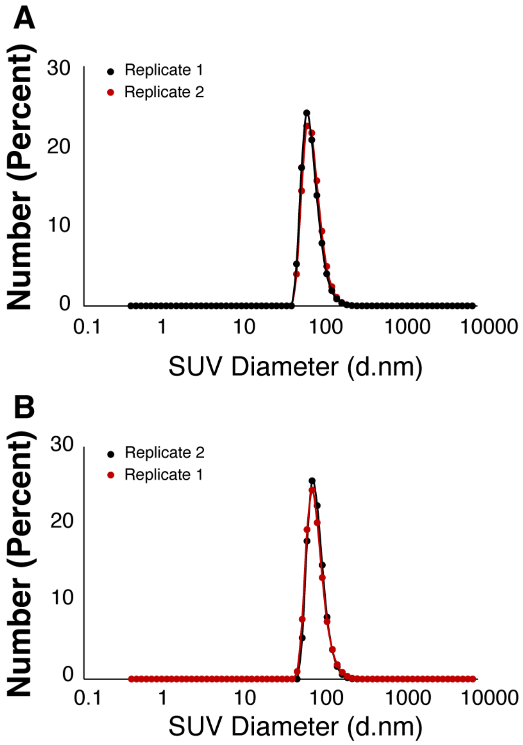

Figure S1. Liposome characterization via dynamic light scattering (DLS). SUVs containing CF-N_3 probe and ascorbic acid were measured in (A) PBS buffer and (B) CuAAC reaction solution.

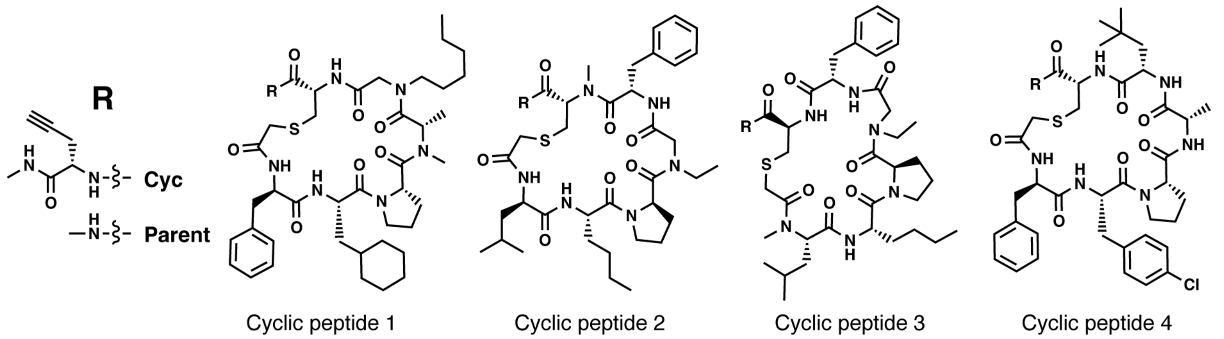

Figure S2. Structures of macrocyclic peptides with and without alkyne labeling.

Table S1. Permeation coefficients of controls, alkyne-tagged macrocycles and their parent molecules.

	MW (Da)	PAMPA P_e (x 10^{-6} cm/s)	PC50 (μM)	Structure
**PEG4s**	296.3	0.008 ± 0.001	N.A.	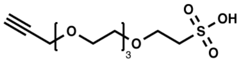
**mPEG17**	774.9	0.004 ± 0.000	N.A.	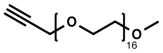
**mPEG8**	378.5	0.15 ± 0.02	120 ± 30	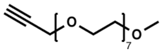
**mPEG4**	202.3	18 ± 1	120 ± 20	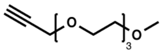
**Cyc1**	893.2	9.3 ± 0.9	7 ± 3	
**Cyc2**	893.5	12.0 ± 0.6	13 ± 8	
**Cyc3**	839.1	6.3 ± 0.7	14 ± 2	
**Cyc4**	839.1	1.5 ± 0.2	58 ± 7	
**Parent1**	798.1	10.3 ± 0.4		
**Parent2**	798.4	9.8 ± 0.7		
**Parent3**	744.0	5.2 ± 0.1		
**Parent4**	744.0	2.9 ± 0.1		

Molecular weight (MW), egg lecithin parallel artificial membrane permeability assay (PAMPA) permeation coefficient (P_e), liposomal permeation assay PC50, red entries classified "impermeable".

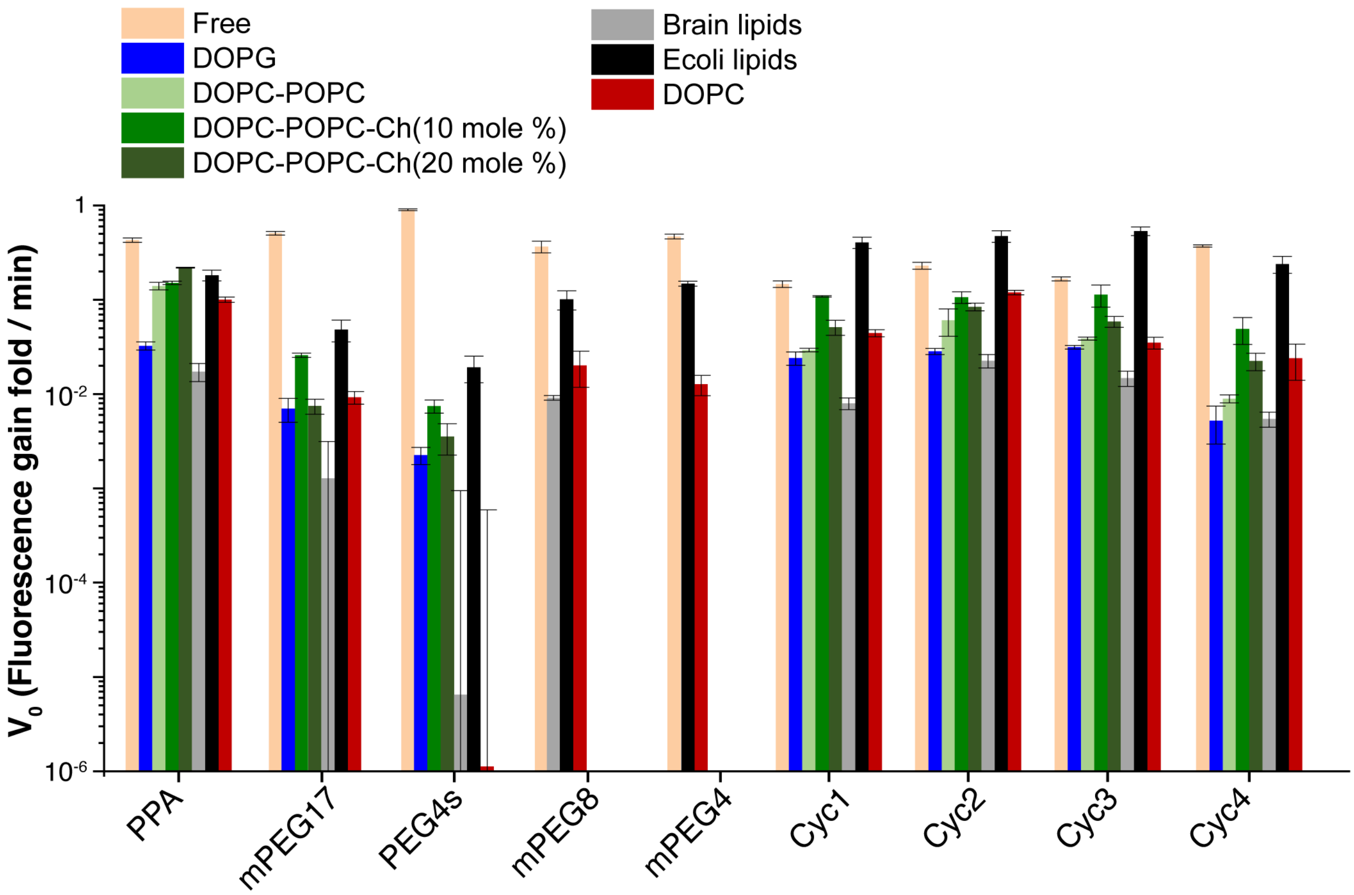

Figure S3. Liposomal permeation assay using different lipid mixtures. Permeabilities of alkyne-tagged cyclic peptides (Cyc1-Cyc4), PPA, mPEG17, PEG4s, mPEG8, and mPEG4 are measured using SUVs composed of DOPG, DOPC, DOPC/POPC, DOPC/POPC/Ch(10 mol% cholesterol), DOPC/POPC/Ch (20 mol% cholesterol), DOPC, brain polar lipids, and *E.coli* polar lipids. CuAAC reaction rates of CF-N_3 probe and sample alkyne are also measured (Free). Initial velocity (V_0) of the CuAAC reaction in liposomal assay was calculated. Error bars indicate the standard error of the mean (N = 3).

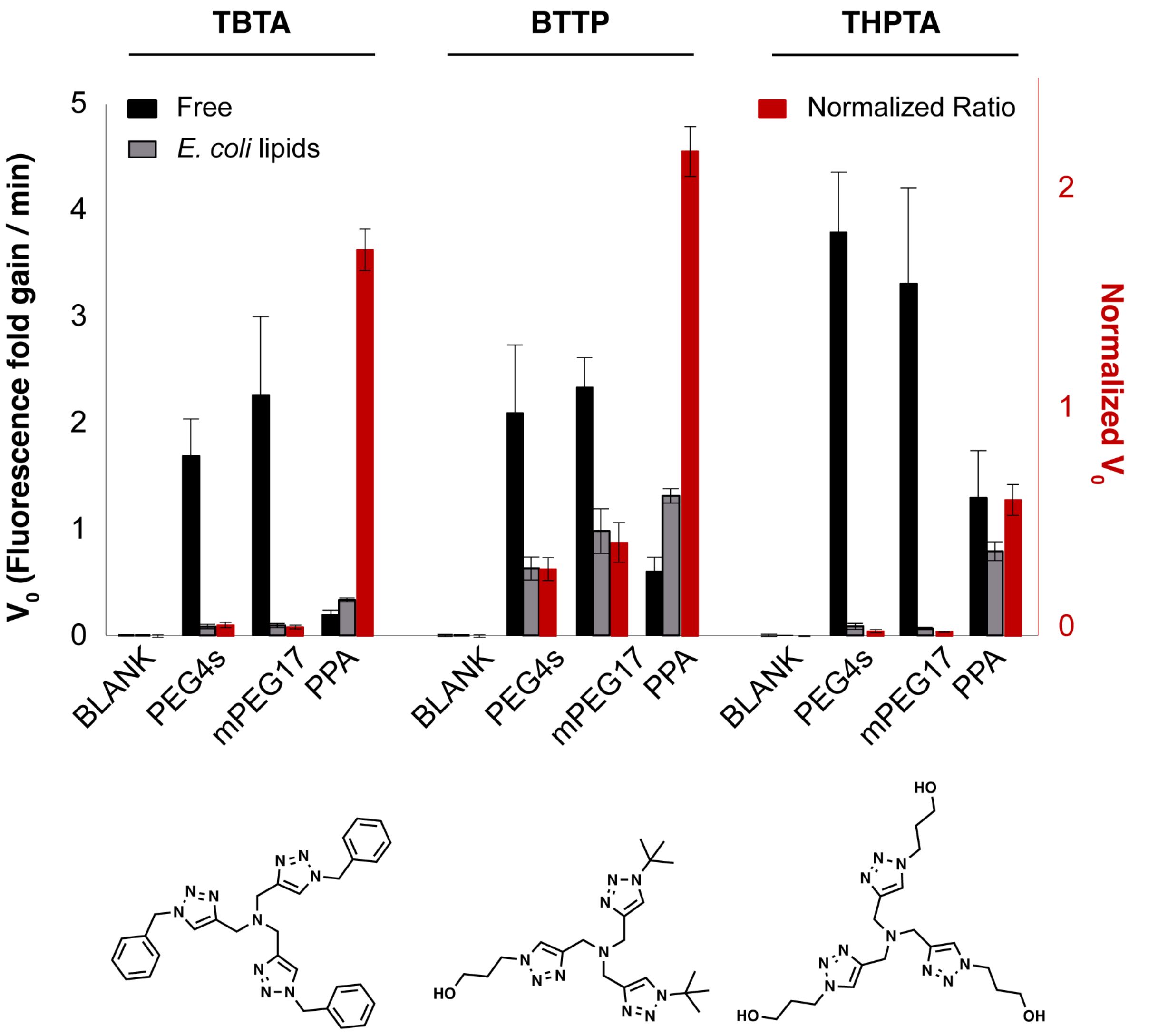

CLogP: 2.7 **CLogP: -0.495** **CLogP: -3.699**

Figure S4. Ligand test for liposomal permeation assay. CuAAC reactions of PEG4s, mPEG17, and PPA with free probe or E. coli extract liposomal probe (20 µM CF-N_3) were conducted with three different ligands (TBTA,BTTP, THPTA) of varying calculated partition coefficients (CLogP). Initial CuAAC reaction velocity (V_0, left axis) was normalized to the blank (no alkyne). CuAAC reaction V_0 in the liposomal assay was normalized to the corresponding V_0 without liposomes (normalized V_0, right axis). Error bars indicate the standard error of the mean (N = 3).

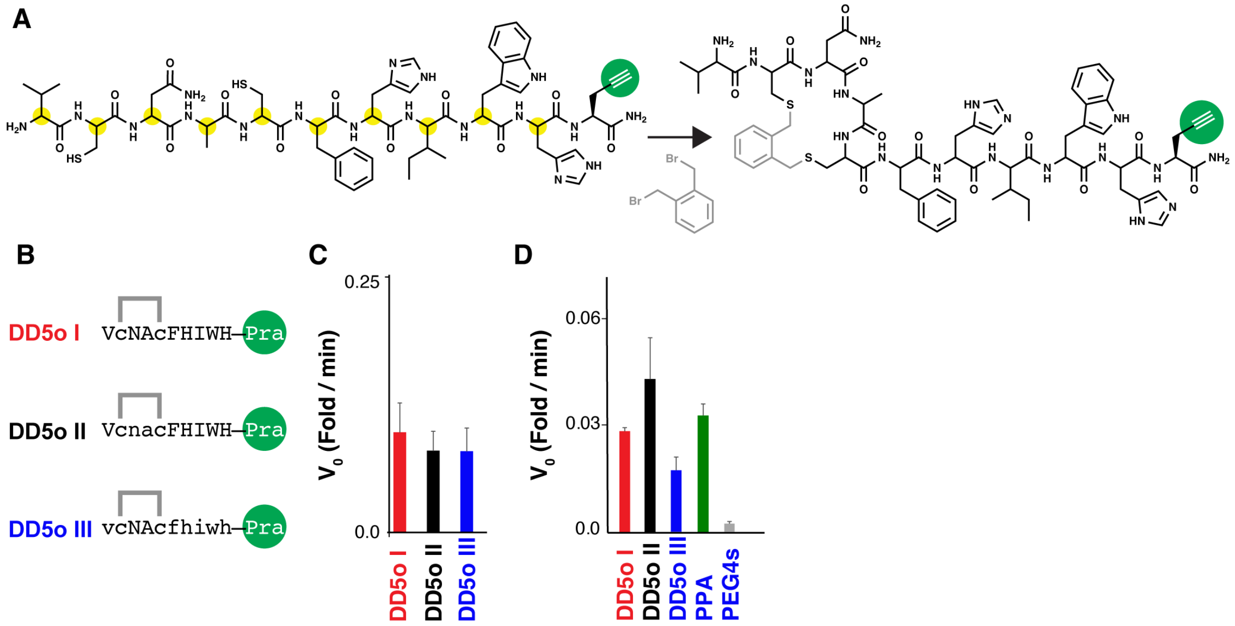

Figure S5. Cell-penetrating peptide design, synthesis, and assay. (A) The peptide backbone displays varied stereocenters (yellow circles). (B) Cell-penetrating peptide (DD5o I, red) and diastereomers (DD5o II, black; DD5o III, blue) are shown with Cys-Cys macrocyclization and C-terminal propargylglycine (Pra). Uppercase: L-amino acid; Lowercase: D-amino acids. (C) Initial CuAAC reaction velocity of DD5o I/II/III with CF-N_3 was measured at 125 µM peptide concentration. Error bars indicate the standard error of the mean (N = 3). (D) Membrane permeability of DD5o I/II/III was detected by liposomal permeation assay using DOPG vesicles with PPA and PEG4s as positive and negative controls, respectively. Error bars indicate the standard error of the mean (N = 3).

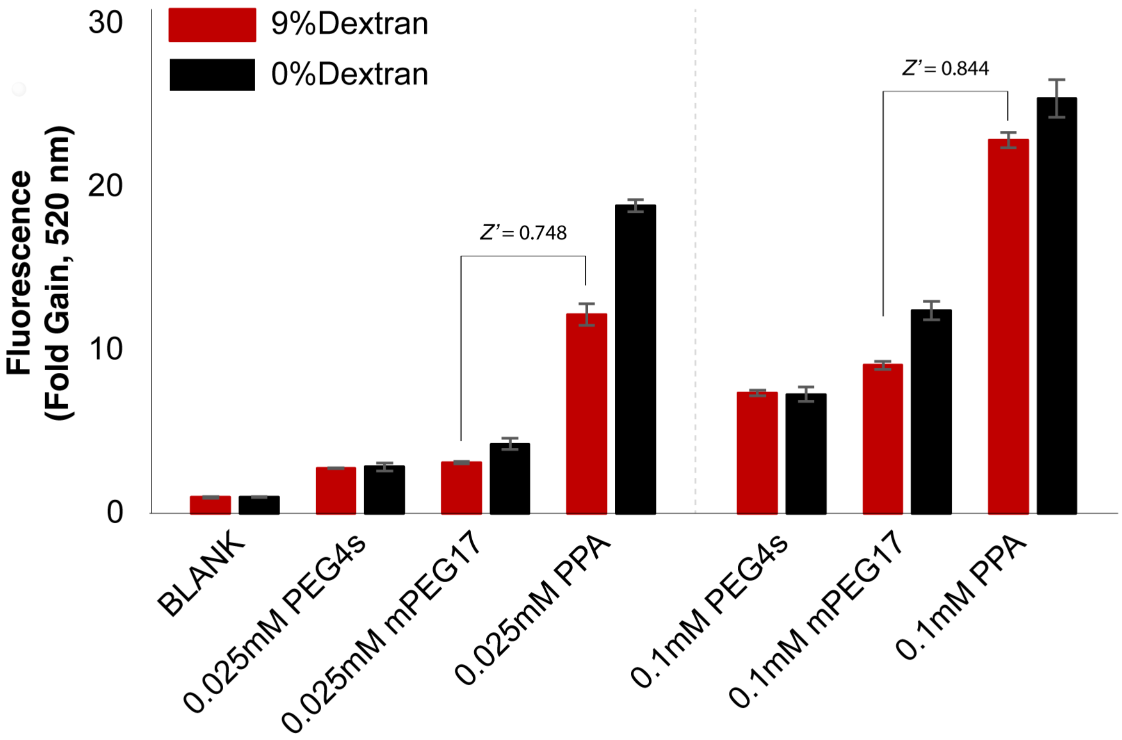

Figure S6. Density additive effect on liposomal permeation assay. Liposomal permeation assay using *E.coli* extract polar lipids with 20 µM CF-N_3 were applied to measure the permeation of PEG4s, mPEG17, and PPA under the condition with or without 9% Dextran. Background-corrected fluorescence (fold gain, 70 min reaction) was calculated. Assay quality (Z′) was calculated for mPEG17 and PPA. Error bars indicate the standard deviation of the mean (N = 3).

A

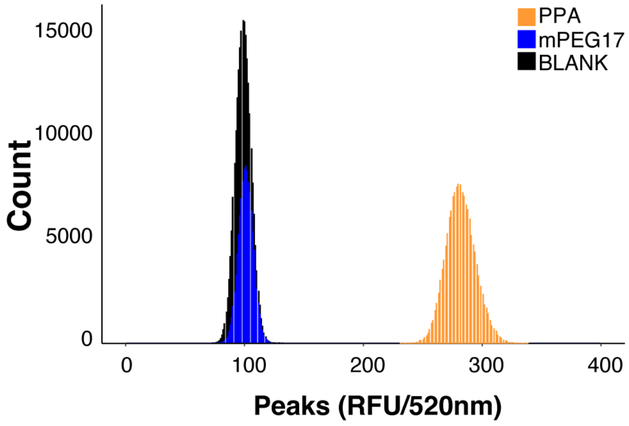

	Mean	SD	*Z'*
mPEG17	96.78	7.18	
PPA	282.20	14.40	0.651

B

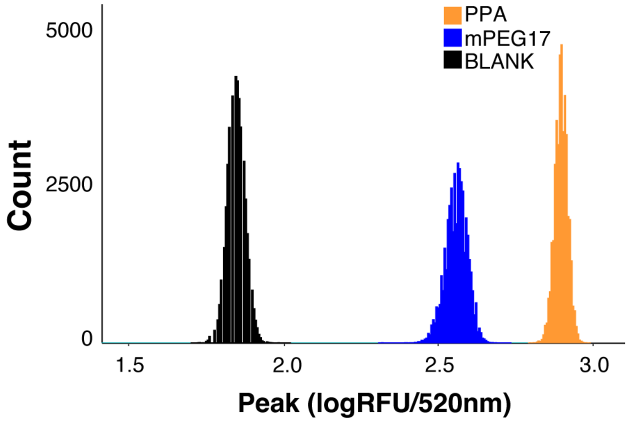

	Mean	SD	*Z'*
mPEG17	367.02	30.13	
PPA	820.29	41.72	0.524

Figure S7. Microfluidic permeation flow injection analysis. (A) Droplet fluorescence populations were acquired for droplets containing PPA or mPEG17 (1 mM), or buffer (BLANK) after on-chip incubation (15 min). (B) The same samples (0.1 mM) combined with density additive (9% dextran) were observed after on-chip incubation (70 min). Assay quality (Z′) was calculated for mPEG17 and PPA populations.

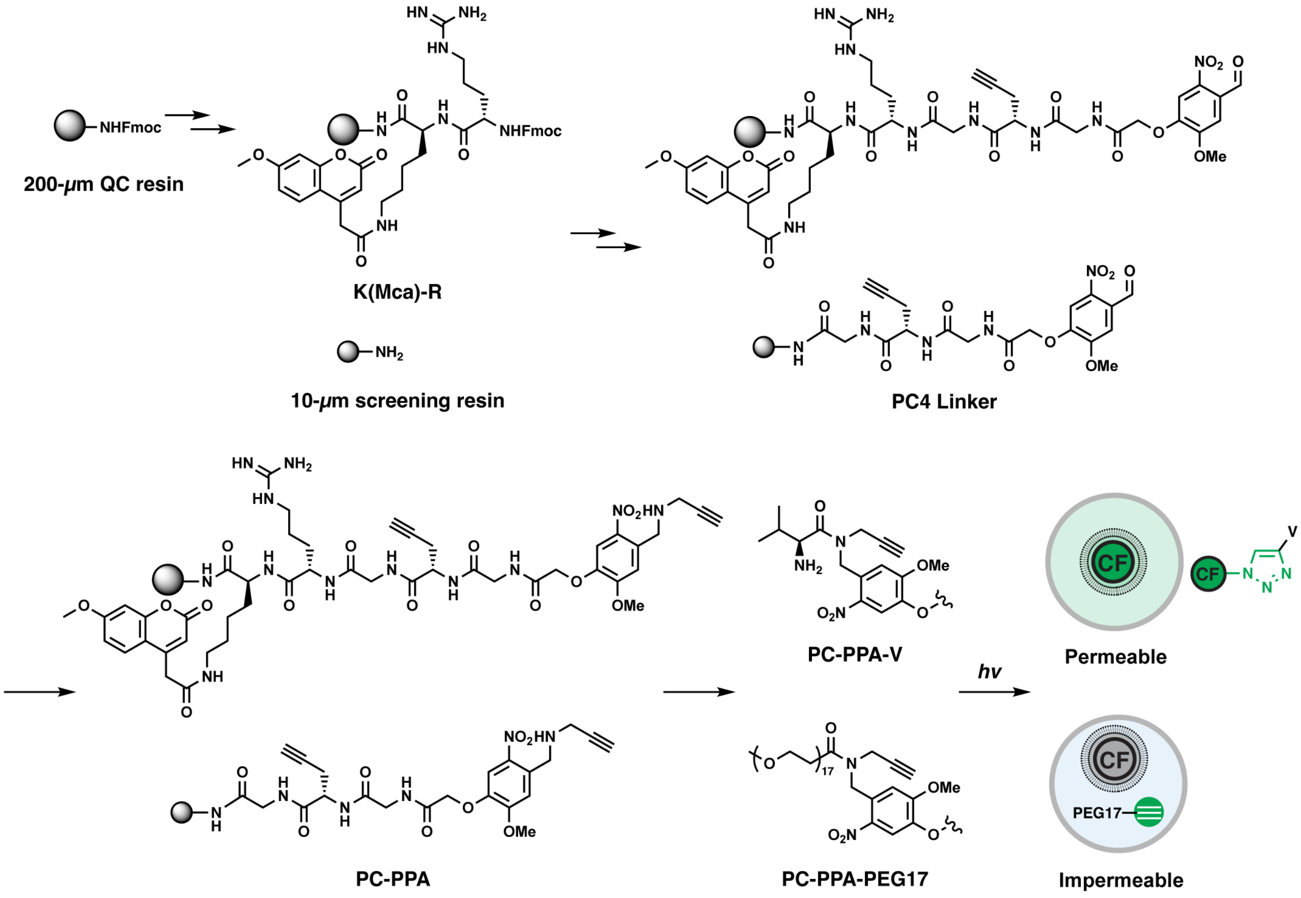

Figure S8. Solid-phase synthesis of PC-PPA-V and PC-PPA-PEG17 tool compound beads. Synthesis was conducted on mixed-scale resin. QC resin (200 µm) was prepared with coumarin K(Mca) spectroscopic handle and arginine ionization flight tag. QC resin is acidic labile Rink Amide resin. QC resin is used for synthesis validation. Screening resin (10 µm) lacks K(Mca) and R residues. Screening resin is used in microfluidic droplet assays and separated from QC resin prior to use. The schematic (lower right) illustrates activation of the liposomal CF-N_3 by photocleaved alkyne-labeled valine (PPA-V) in the microfluidic droplet, and no permeation and subsequent liposomal probe activation with photocleaved PPA-PEG17.

Table S2. MALDI-TOF MS analysis of microcleavage products from QC resins.

	Structure	Exact Mass	Expected Mass [M+H]$^+$
K(Mca)-R	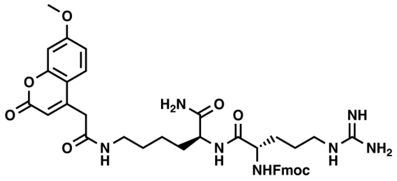	739.33	740.3
PC4 Linker	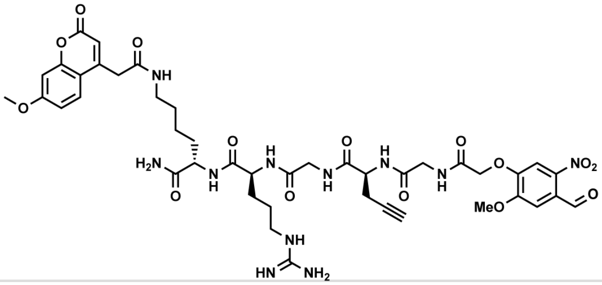	963.37	964.4
PC-PPA	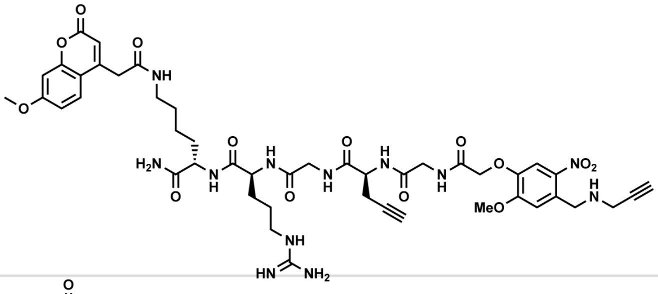	1002.42	1003
PC-PPA-PEG17	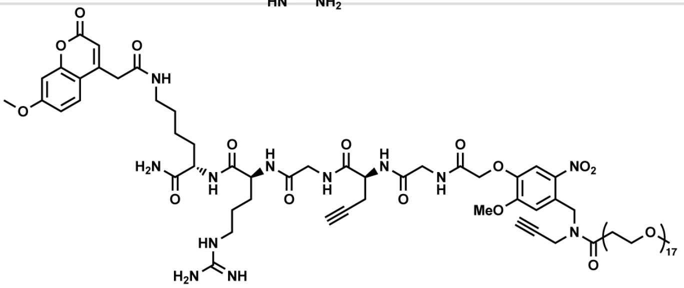	1792.88	1793
PC-PPA-V	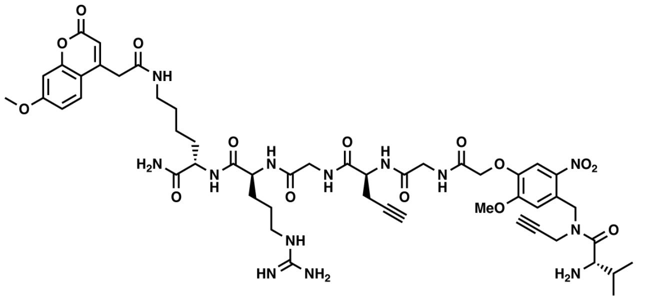	1101.49	1102

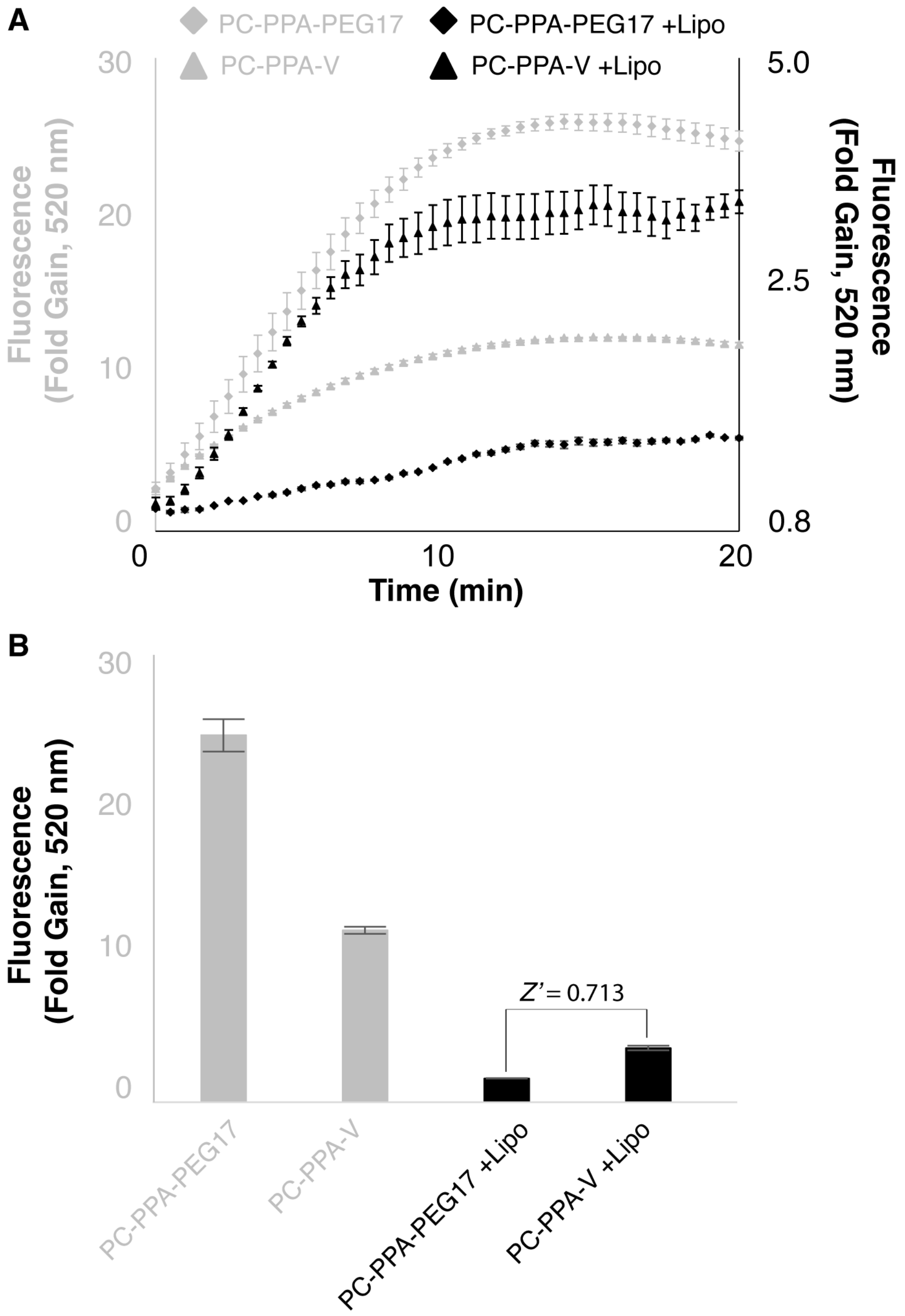

Figure S9. Liposomal permeation assay of photocleaved control compounds. (A) Fluorogenic CuAAC reaction progress with CF-N_3 (gray) and liposomal CF-N_3 (+Lipo, black) using PC-PPA-V and PC-PPA-PEG17 photocleaved control compounds is shown. CF-N_3 (20 µM) was encapsulated in SUVs composed of *E. coli* extract polar lipids. Error bars indicate the standard error of the mean (N = 3). (B) Fluorescence fold gain at 20-min end point was calculated for Z-factor evaluation. Error bars indicate the standard deviation of the mean (N = 3).

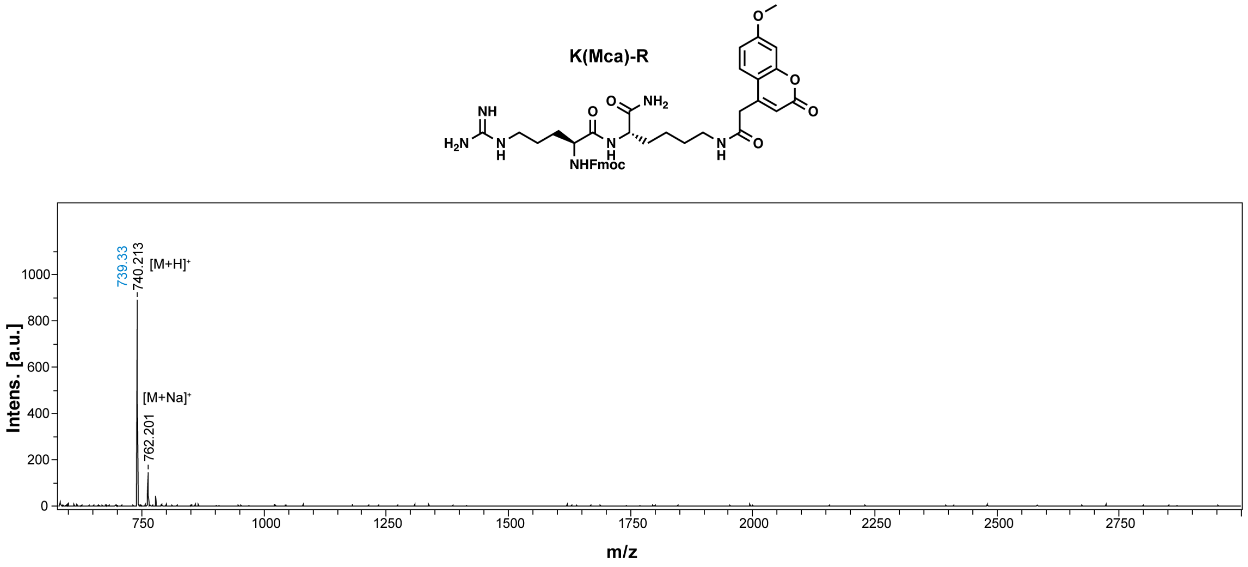

Figure S10. MALDI-TOF MS analysis confirmed the presence of K(Mca)-R, the microcleavage product from QC resins. The mass spectrum displays the theoretical exact mass (cyan) and observed mass (black) for $[M+H]^+$ and $[M+Na]^+$.

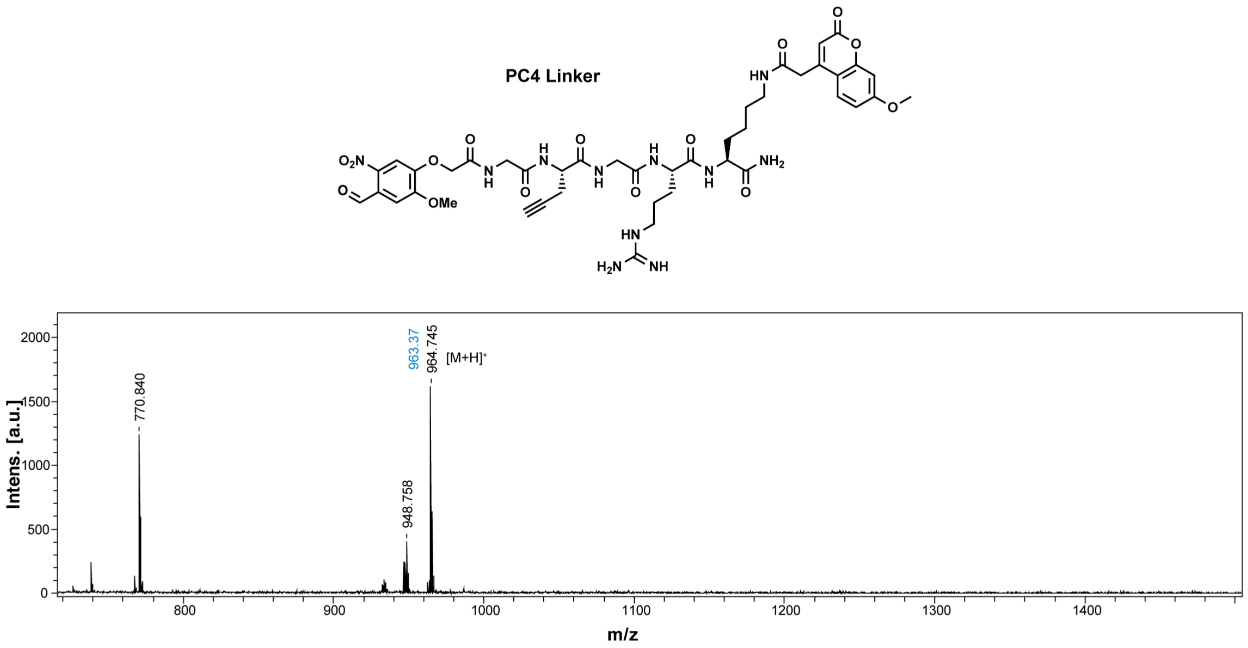

Figure S11. MALDI-TOF MS analysis confirmed the presence of PC4 linker, the microcleavage product from QC resins. The mass spectrum displays the theoretical exact mass (cyan) and observed mass (black) for $[M+H]^+$.

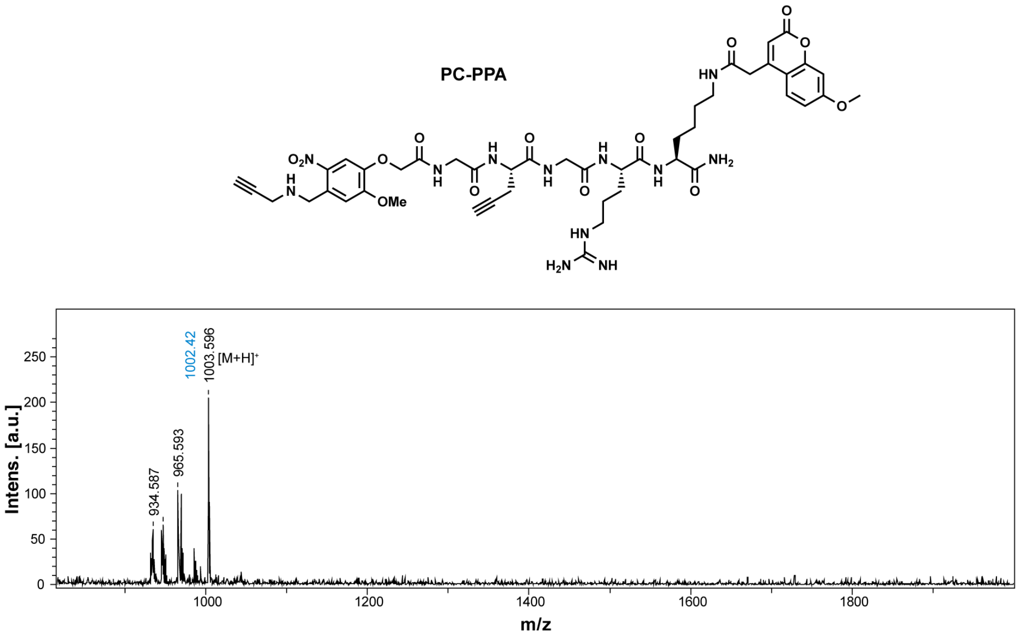

Figure S12. MALDI-TOF MS analysis confirmed the presence of PC-PPA, the microcleavage product from QC resins. The mass spectrum displays the theoretical exact mass (cyan) and observed mass (black) for $[M+H]^+$.

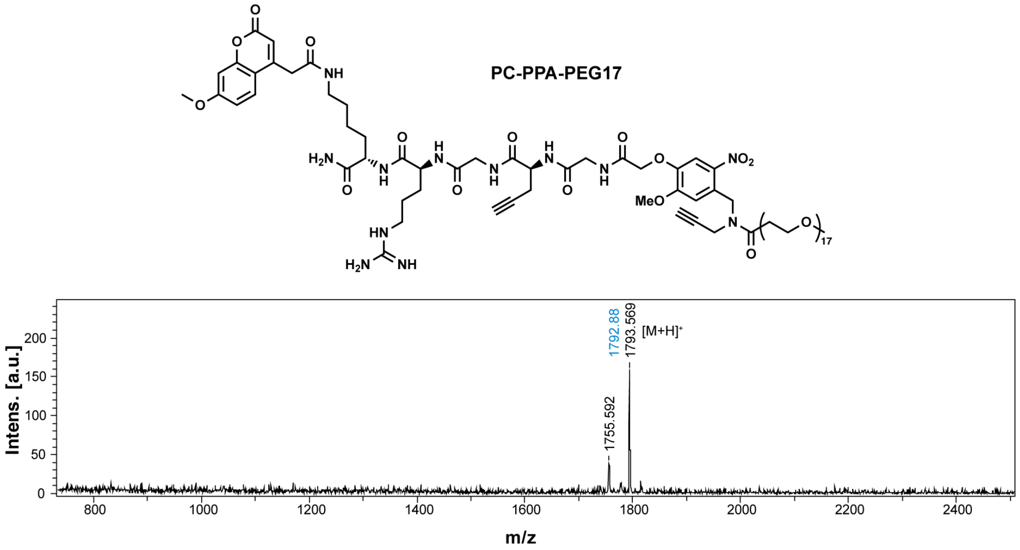

Figure S13. MALDI-TOF MS analysis confirmed the presence of PC-PPA-PEG17, the microcleavage product from QC resins. The mass spectrum displays the theoretical exact mass (cyan) and observed mass (black) for $[M+H]^+$.

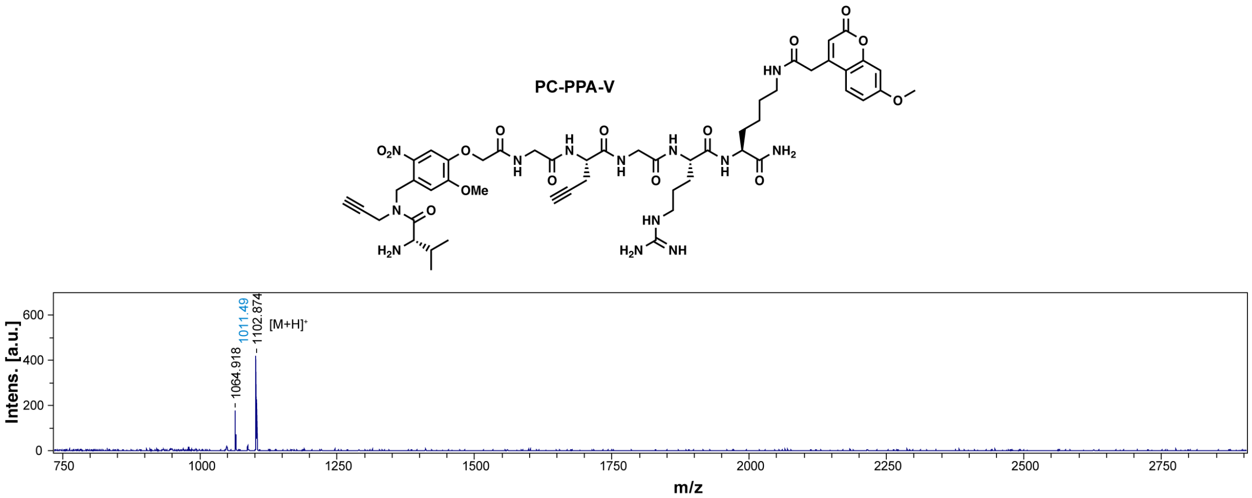

Figure S14. MALDI-TOF MS analysis confirmed the presence of PC-PPA-V, the microcleavage product from QC resins. The mass spectrum displays the theoretical exact mass (cyan) and observed mass (black) for $[M+H]^+$.

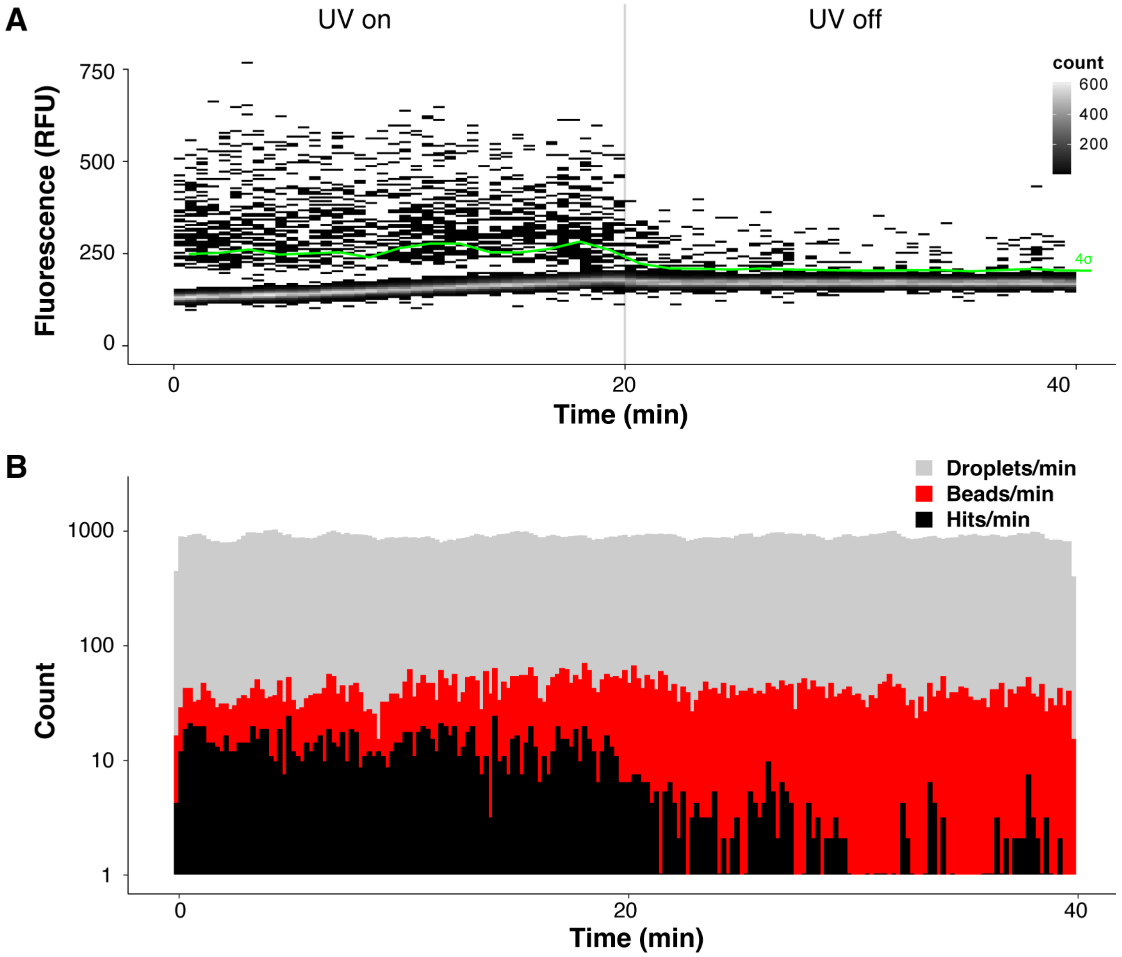

Figure S15. Microfluidic photodosing control experiment. (A) PC-PPA-V beads are loaded in microfluidic droplets of liposomal permeation assay (*E. coli* extract polar lipid SUVs, 20 μM CF-N_3). Droplet fluorescence was measured with or without UV irradiation in flow (UV on or UV off, respectively). A simulated sort threshold 4 standard deviations (4σ) above the mean negative signal is indicated (green line). (B) The numbers of total droplets (gray), droplets with beads (red), and hit droplets (black) are plotted.

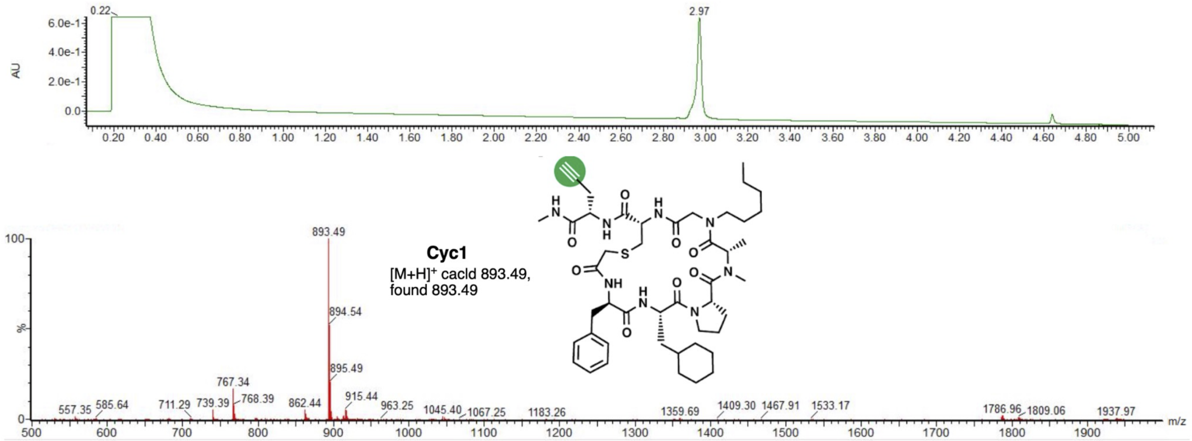

Figure S16. LC-MS analysis of macrocyclic peptide Cyc1.

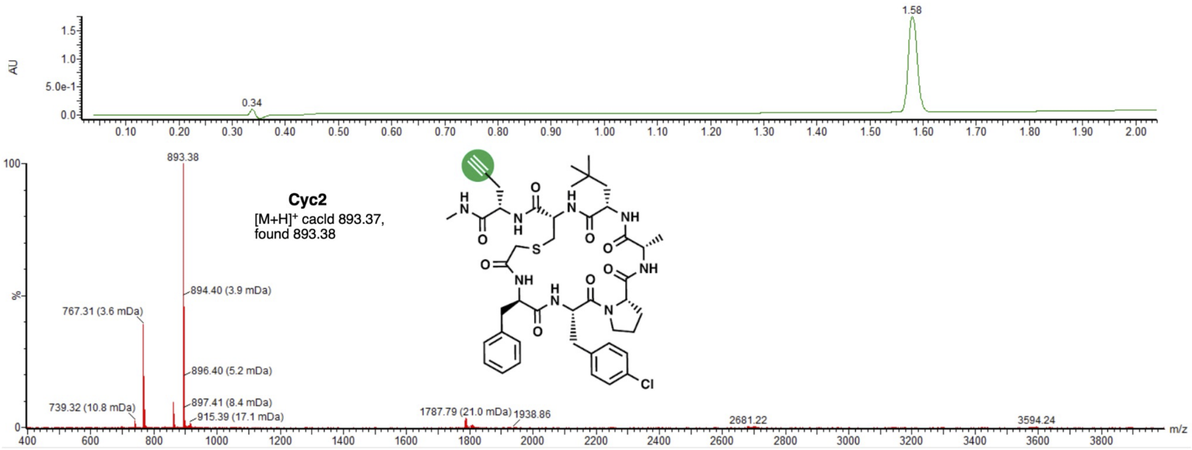

Figure S17. LC-MS analysis of macrocyclic peptide Cyc2.

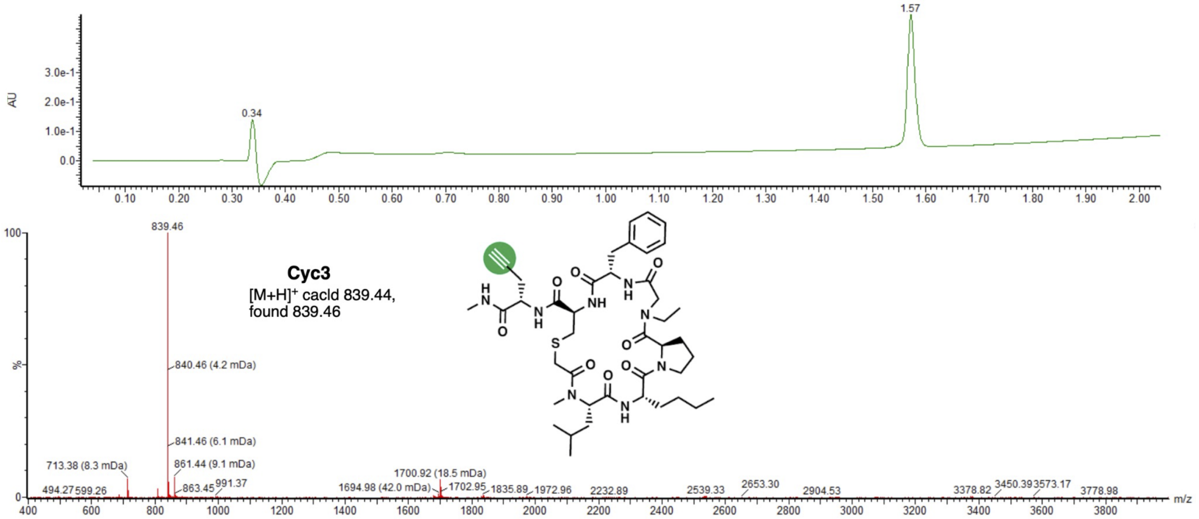

Figure S18. LC-MS analysis of macrocyclic peptide Cyc3.

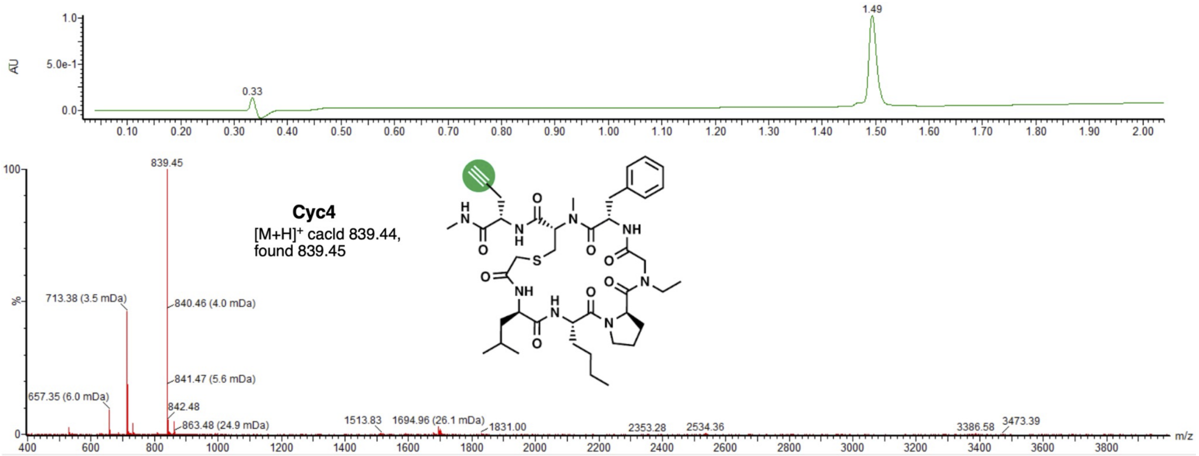

Figure S19. LC-MS analysis of macrocyclic peptide Cyc4.

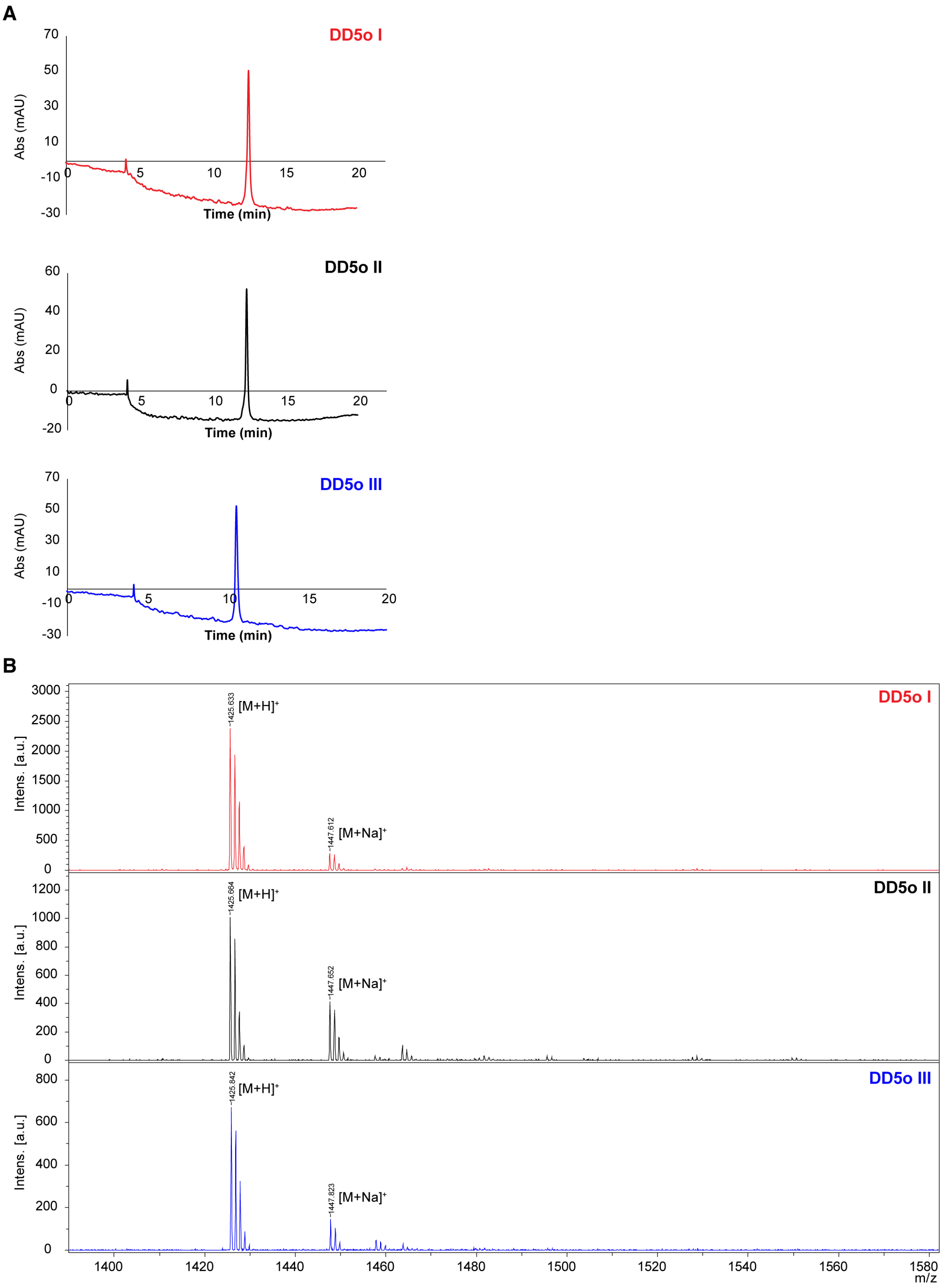

Figure S20. Characterization of DD5o I/II/III peptides. (A) HPLC analysis indicated the presence of a single major product peak. (B) MALDI-TOF MS analysis of the major product HPLC fraction confirmed the presence of DD5o I/II/III $[M+H]^+$.
